# Efficacy and Safety of Ablation for Symptomatic Atrial Fibrillation in Elderly Patients: A Meta-Analysis

**DOI:** 10.3389/fcvm.2021.734204

**Published:** 2021-09-20

**Authors:** Wei-Chieh Lee, Po-Jui Wu, Huang-Chung Chen, Hsiu-Yu Fang, Ping-Yen Liu, Mien-Cheng Chen

**Affiliations:** ^1^College of Medicine, Institute of Clinical Medicine, National Cheng Kung University, Tainan, Taiwan; ^2^Division of Cardiology, Department of Internal Medicine, Kaohsiung Chang Gung Memorial Hospital, Chang Gung University College of Medicine, Kaohsiung, Taiwan; ^3^Division of Cardiology, Department of Internal Medicine, College of Medicine, National Cheng Kung University Hospital, National Cheng Kung University, Tainan, Taiwan

**Keywords:** elderly, atrial fibrillation, radiofrequency ablation, cryoballoon ablation, recurrence

## Abstract

**Background:** Age affects the efficacy of pharmacological treatment for atrial fibrillation (AF). Catheter ablation, including radiofrequency (RF) or cryoballoon ablation, is an effective strategy for symptomatic AF. This meta-analysis aimed to analyze the efficacy and safety of AF ablation in elderly patients with AF compared to non-elderly patients with AF.

**Methods:** We searched several databases for articles published between January 1, 2008 and March 31, 2020. Eighteen observational studies with 21,039 patients were analyzed. Data including recurrence of AF or atrial tachyarrhythmia (ATA), complications, procedural time, and fluoroscopic time were compared between the elderly and non-elderly groups.

**Results:** The elderly patients had significantly higher incidences of recurrent AF or ATA after AF ablation compared to the non-elderly patients (<60 years old) (odds ratio [OR], 1.21; 95% confidence interval [CI], 1.11–1.33). The elderly patients had significantly higher incidences of complications of AF ablation compared to the non-elderly patients (OR, 1.37; 95% CI, 1.14–1.64). However, elderly AF patients with age ≥75 years old had similar incidence of recurrent AF or ATA and complication after AF ablation compared to non-elderly patients with AF.

**Conclusions:** The elderly patients had significantly higher incidences of recurrent AF or ATA and complications after ablation for non-paroxysmal AF compared to non-elderly patients with AF (<60 years old), except in patients ≥75 years old.

## Introduction

As the elderly population grows and the quality of the healthcare system improves, the burden of treating elderly patients with atrial fibrillation (AF) increases gradually (1, 2). However, higher ischemic risks and less effectiveness of antiarrhythmic medications are expected in elderly patients with AF (3, 4). Therefore, there is still a big challenge for physicians to treat elderly patients with AF. In addition, the elderly patients with AF tend to have a large left atrium with electrical and structural remodeling and fibrosis, which also reduce the efficacy of pharmacological treatment (5, 6). Catheter ablation has recently emerged as an important therapeutic strategy to achieve and maintain a normal sinus rhythm in symptomatic patients with AF (7, 8). Moreover, catheter ablation for AF has been reported to reduce mortality and HF readmission in patients with heart failure (HF) (9). The prevalence of HF was higher in the elderly population than in the younger population (10). However, the efficacy and safety of catheter ablation in elderly patients with AF have not been clearly explored. Previous studies comparing the outcomes of AF ablation between elderly and non-elderly populations had different criteria for elderly or different age distribution and had inconsistent results. This study aimed to explore the efficacy and safety of AF ablation in elderly patients with AF compared to non-elderly patients with AF.

## Methods

### Search Strategies, Trial Selection, Quality Assessment, Review Process, and Data Extraction

**Figure 1:**
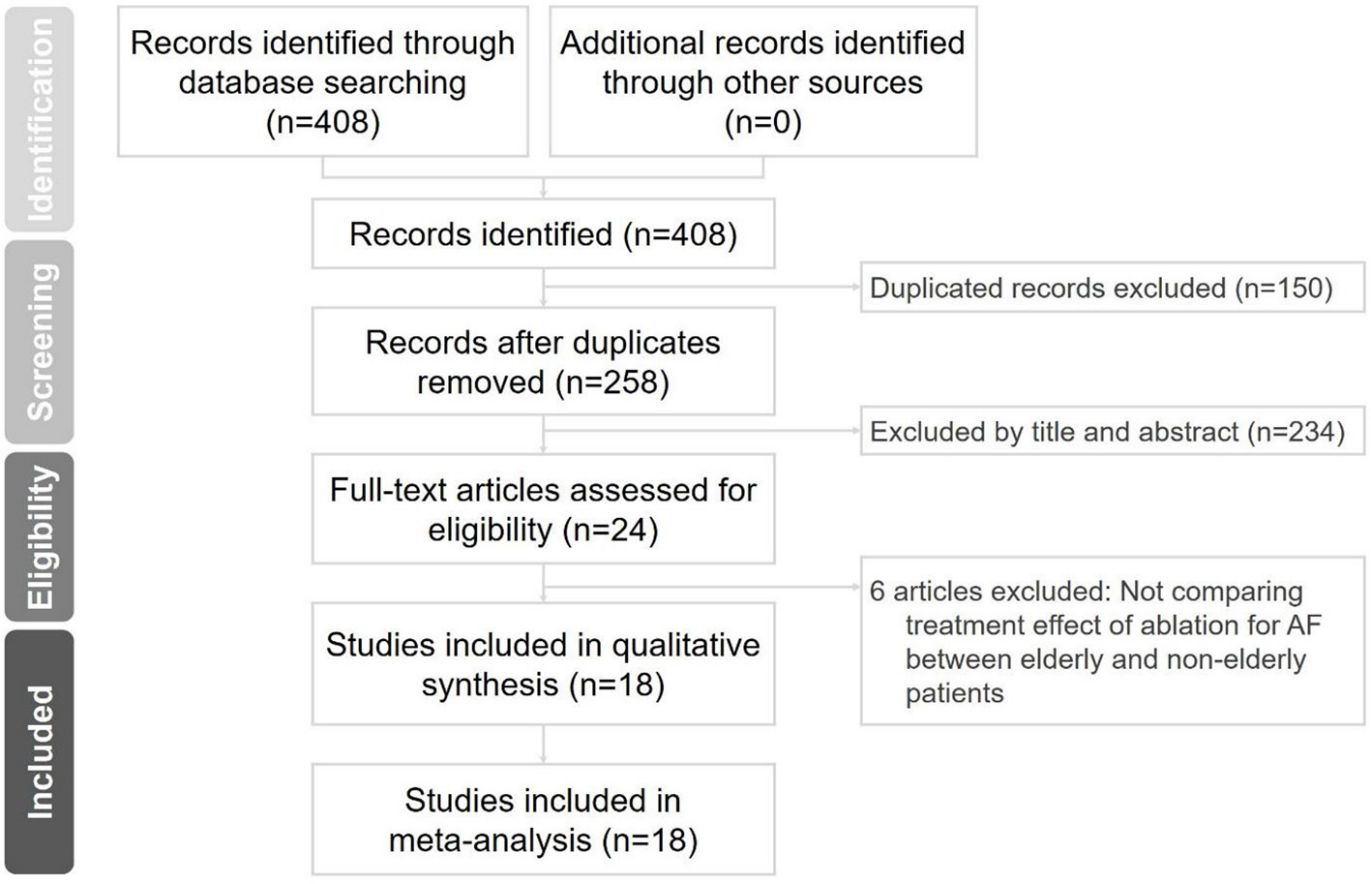
Flowchart of the selection strategy and inclusion and exclusion criteria for this meta-analysis. AF, atrial fibrillation.

Systematic literature searches for published articles between January 1, 2008 and December 31, 2020, in PubMed, Embase, Cochrane Library, ProQuest, ScienceDirect, ClinicalKey, Web of Science, and ClinicalTrials.gov were separately performed by two cardiologists. The keywords “elderly,” “atrial fibrillation ablation,” “radiofrequency ablation,” “cryoballoon ablation,” and “efficacy” were used. We did not set language restrictions to increase the number of eligible articles, and disagreements were resolved by a third reviewer. Only randomized controlled trials and clinical studies that compared the clinical outcomes between elderly and non-elderly groups of different age distributions after non-valvular AF ablation were included in the present meta-analysis. The inclusion criteria were human studies with a parallel design. The exclusion criteria included conference abstracts, case reports or series, animal studies, and review articles. Figure 1 illustrates the literature search and screening protocol. Our search identified 258 articles after removing duplicates. Among them, 18 observational and cohort studies with 21,039 participants met our inclusion criteria and were included in this study.

### Assessment of Risk of Bias in the Included Studies

The risk of bias in the included studies was appraised by two independent reviewers (WC Lee and PJ Wu) according to the Risk Of Bias In Non-Randomized Studies Of Interventions (ROBINS-I) tool, with disagreements resolved by consensus or by arbitration with a third author (HY Fang). The ROBINS-I requires the assessment of the following domains: bias due to confounding, bias in selection of participants into the study, bias in measurement of exposure, bias in Departures from exposure, bias due to missing data, bias in measurement of the outcome, and bias in selection of the reported result.

### Statistical Analysis

All analyses were performed using the Comprehensive Meta-Analysis software, version 3 (Biostat Inc., Englewood, NJ, USA) and Cochrane RevMan software (version 5.4.1). The frequency of each evaluated outcome was extracted from each study and was presented as the cumulative rate. The standardization of each evaluated result originated from each study was presented as standardized mean differences (SMDs). A random-effects model was used to pool individual odds ratios (ORs). The chi-square test was used to evaluate heterogeneity across trials (*p* ≤ 0.1, considered significant). *I*^2^ statistics (>50% was considered significant heterogeneity) was used to examine each outcome. Funnel plots and Egger's test were used to access potential publication bias (*p* ≤ 0.1 was considered significant). Statistical significance was set at *p* < 0.05.

## Results

### Characteristics of Included Studies

The study selection process is displayed in Figure 1, and 18 studies met the inclusion criteria. In total, 21,039 participants were included. The definition of elderly in each study, participant characteristics, study period, and ablation method are shown in Table 1. The definition of elderly or age distribution differed between enrolled studies including those aged ≥60 (11, 12), ≥65 (13–16), ≥70 (17, 18), ≥75 (19–24), and ≥80 (25–28) years. One study with RF ablation and cryoballoon ablation was excluded in the analysis for ablation method and procedural complications (16).

**Table 1:** Characteristics of the 18 included studies.

**The definition of elderly**	**First author (year)**	**Patients number (male %)**	**Age (years)**	**Study period**	**The prevalence of PAf (%)**	**Study design**	**Ablation method**
≥60 years old	Bhargava et al. (11)	323 (80.2)	54 ± 12	N/A	53.8	Cohort study	RF ablation
	Liu et al. (12)	7,926 (70.0)	56 ± 10	1998–2009	80.0	Cohort study	RF ablation
≥65 years old	Leong-Sit et al. (13)	1,548 (76.8)	56 ± 22	2000/11–2008/9	64.8	Cohort study	RF ablation
	Guiot et al. (14)	1,016 (71.7)	62 ± 11	2001–2009	60.3	Cohort study	RF ablation
	Lioni et al. (15)	316 (56.3)	57 ± 12	N/A	100	Cohort study	RF ablation
	Kis et al. (16)	390 (73.1)	N/A (20.5% >65 y/o)	2001/3–2011/12	90.3	Cohort study	RF ablation or cryoballoon ablation
≥70 years old	Traub et al. (17)	60 (73.3)	58 ± 14	2003/2–2007/2	100	Cohort study	RF ablation
	Kautzner et al. (18)	3,197 (68.2)	59 ± 10	2001/1–2016/12	77.6	Cohort study	RF ablation
≥75 years old	Zado et al. (19)	1165 (77.3)	55 ± 11	2000/11–2007/7	64.0	Cohort study	RF ablation
	Kusumoto et al. (20)	240 (72.1)	66 ± 10	2004/12–2006/12	62.1	Cohort study	RF ablation
	Abugattas et al. (21)	159 (54.1)	65 ± 12	2012/6–2016/2	100	Cohort study	Cryoballoon ablation
	Tscholl et al. (22)	80 (57.5)	75 ± 12	N/A	46.3	Cohort study	Cryoballoon ablation
	Abdin et al. (23)	238 (60.9)	65 ± 11	2015/7–2017/3	38.2	Cohort study	Cryoballoon ablation
	Heeger et al. (24)	208 (51.0)	70 ± 10	N/A	57.2	Cohort study	Cryoballoon ablation
≥80 years old	Bunch et al. (25)	752 (58.6)	65 ± 11	2005/3–2008/5	53.7	Cohort study	RF ablation
	Tan et al. (26)	377 (N/A)	72 ± 8	2006/1–2007/10	42.2	Cohort study	RF ablation
	Santangeli et al. (27)	2,754 (69.3)	63 ± 22	2008–2011	28.9	Cohort study	RF ablation
	Kanda et al. (28)	290 (57.9)	69 ± 11	N/A	100	Cohort study	Cryoballoon ablation

*PAf, paroxysmal atrial fibrillation; RF, radiofrequency; N/A, not available*.

### Risk of Bias Assessment

The risk of bias of included studies, according to the ROBINS-I tool, was moderate in eight studies (11, 13, 14, 16, 17, 19, 24, 28), serious in six studies (15, 21–23, 25, 27), and critical in four investigation (12, 18, 20, 26) (Supplementary Table 1).

### Patient Demographics

Table 2 describes the basic demographics and comorbidities of study patients. The elderly group was older (elderly group vs. non-elderly group; 68.7 ± 6.8 years vs. 56.3 ± 13.3 years, *p* < 0.001) and had fewer male patients (elderly group vs. non-elderly group; 61.0 vs. 72.2%, *p* < 0.001). The elderly group had significantly higher prevalence of diabetes mellitus, hypertension, heart failure, coronary artery disease, and paroxysmal AF compared to the non-elderly group.

**Table 2:** Patient demographics.

	**Elderly (5,054)**	**Non-elderly (15,985)**	***p*-value**
Age (years)	68.7 ± 6.8 (4,666)	56.3 ± 13.3 (14,435)	<0.001
Male sex, % (number)	61.0 (3,053)	72.2 (11,306)	<0.001
Diabetes mellitus % (number)	13.1 (174)	10.4 (905)	0.003
Hypertension % (number)	64.6 (979)	47.2 (4,877)	<0.001
Heart failure % (number)	12.9 (159)	10.7 (1,018)	0.020
Coronary artery disease % (number)	17.5 (268)	9.4 (741)	<0.001
Paroxysmal AF % (number)	79.0 (3,995)	64.8 (10,352)	<0.001

*Data are expressed as mean ± standard deviation or as percentage (number). AF, atrial fibrillation*.

### Pooled Odds Ratio of Recurrent Atrial Fibrillation or Atrial Tachyarrhythmia After Ablation in the Elderly vs. Non-elderly Groups

**Figure 2:**
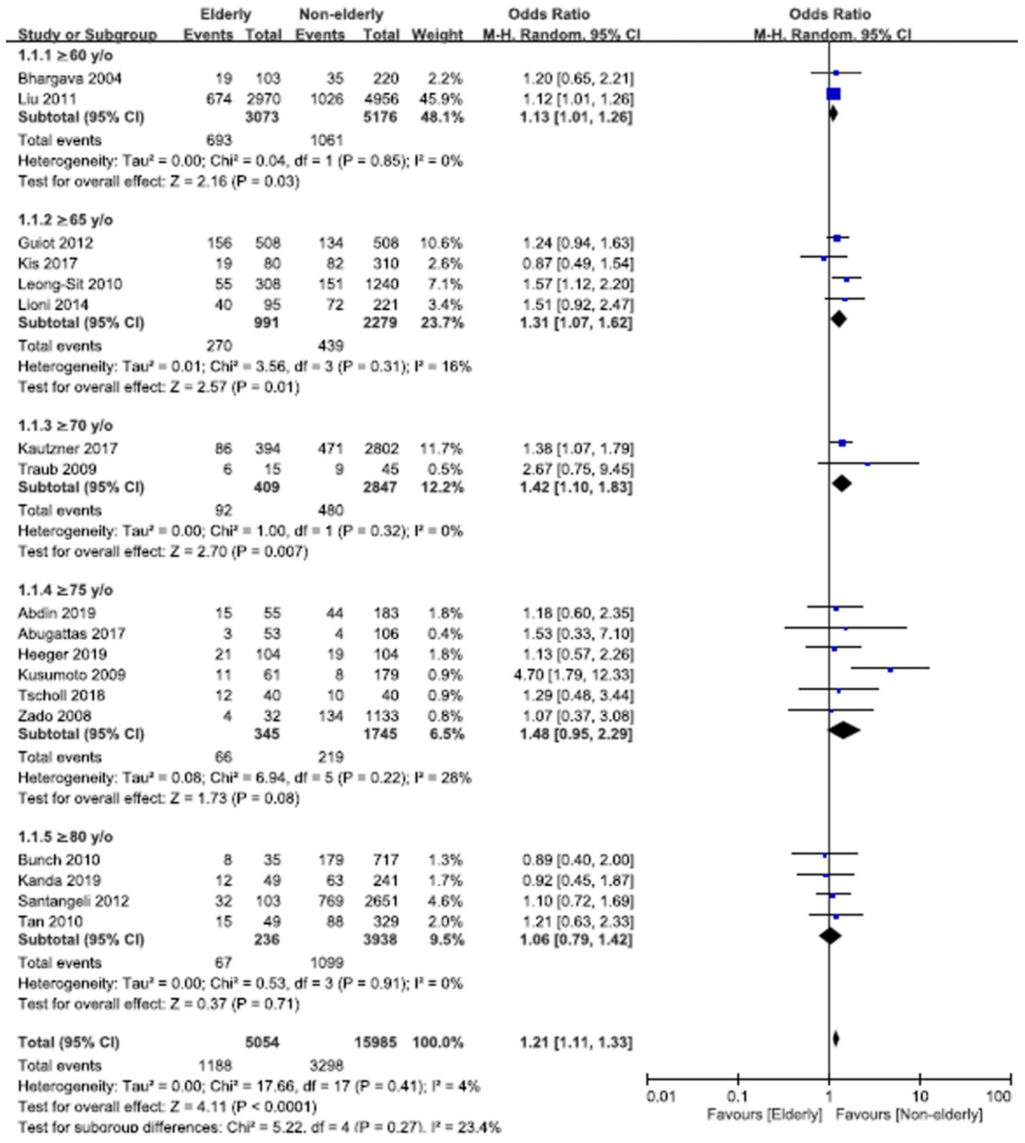
Forest plots of the overall odds ratio (OR) of recurrent atrial fibrillation (AF) or atrial tachyarrhythmia (ATA) after AF ablation between the elderly and non-elderly groups in the entire population with different age distributions from 18 studies. CI, confidence interval.

The overall OR of recurrent AF or atrial tachyarrhythmia (ATA) after ablation in the elderly vs. non-elderly groups was 1.21 (95% confidence interval [CI], 1.11–1.33; Figure 2), with non-significant heterogeneity (Chi^2^, 17.66; *df*, 17; *I*^2^, 4%; *p* = 0.41) and non-significant publication bias according to Egger regression (*t*, 1.33; *df*, 16; *p* = 0.20) on inspection of the funnel plot (Supplementary Figure 1). In the subgroup analysis of age ≥60, ≥65, ≥70, ≥75, and ≥80 years, the ORs (95% CI) of recurrent AF or ATA after ablation in the elderly vs. non-elderly groups were 1.13 (1.01–1.26), 1.31 (1.07–1.62), 1.42 (1.10–1.83), 1.48 (0.95–2.29), and 1.06 (0.79–1.42), respectively. There was non-significant heterogeneity in the subgroups of patients aged ≥60, ≥65, ≥70, ≥75, and ≥80 years. There was no publication bias according to Egger regression on inspection of the funnel plot in the subgroups of age ≥60 (*t*, 0.44; *df*, 2; *p* = 0.71), ≥75 (*t*, 0.60; *df*, 4; *p* = 0.58), and ≥80 years (*t*, 1.02; *df*, 2; *p* = 0.42) (Supplementary Figures 2–4).

### Pooled Odds Ratio of Recurrent Atrial Fibrillation or Atrial Tachyarrhythmia in Different Subgroups in Terms of Ablation Methods and Types of AF

**Figure 3:**
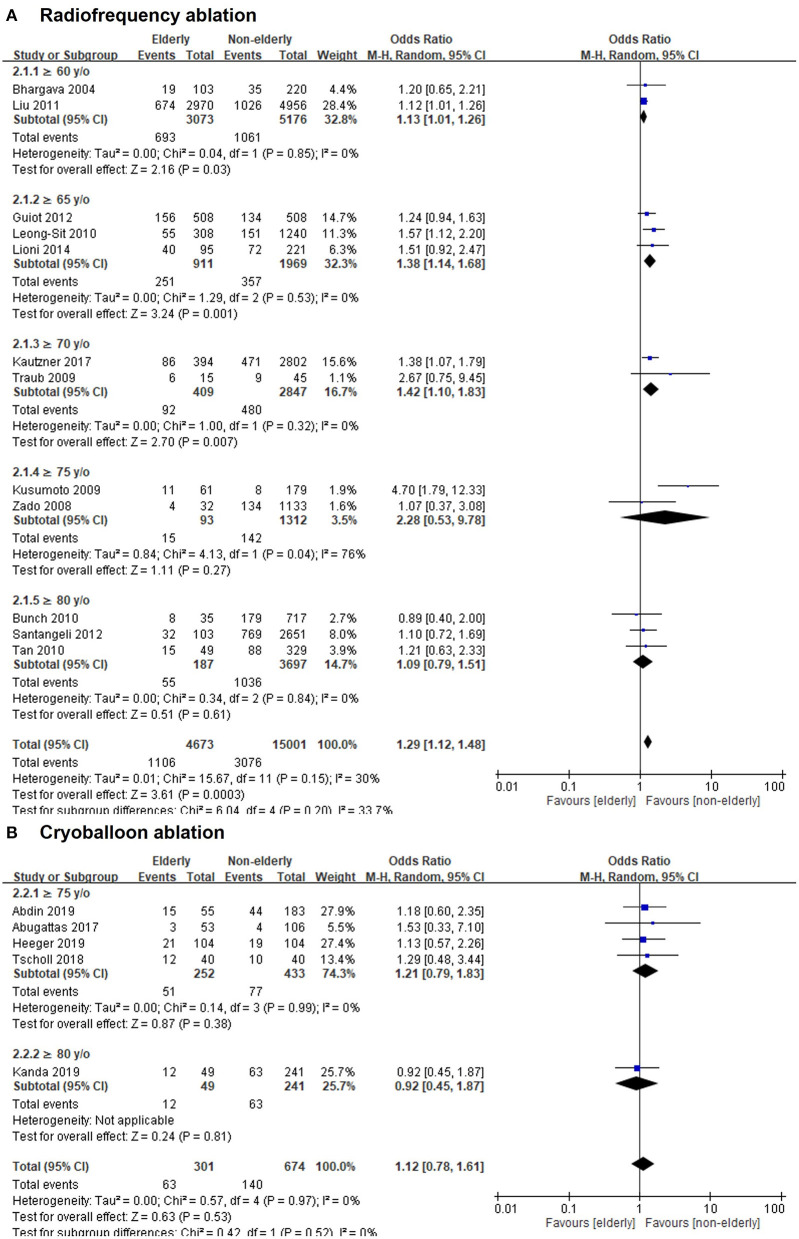
Forest plots of the OR of recurrent AF or ATA after radiofrequency (RF) ablation **(A)** or cryoballoon ablation **(B)** between elderly and non-elderly groups from 17 studies with different age stratification.

In terms of different ablation methods, the OR of recurrent AF or ATA after radiofrequency (RF) ablation in the elderly vs. non-elderly group was 1.29 (95% CI, 1.12–1.48; Figure 3A), with non-significant heterogeneity (Chi^2^, 15.67; *df*, 11; *I*^2^, 30%; *p* = 0.15) but significant publication bias according to Egger regression (*t*, 1.96; *df*, 10; *p* = 0.08) on inspection of the funnel plot (Supplementary Figure 5). In the subgroup analysis of age ≥60, ≥65, ≥70, ≥75, and ≥80 years, the ORs (95% CI) of recurrent AF or ATA after RF ablation in the elderly vs. non-elderly groups were 1.13 (1.01–1.26), 1.38 (1.14–1.68), 1.42 (1.10–1.83), 2.28 (0.53–9.78), and 1.09 (0.79–1.51), respectively. There was no publication bias according to Egger regression on inspection of the funnel plot in the subgroups of age ≥65 (*t*, 0.88; *df*, 1; *p* = 0.54) and ≥80 years (*t*, 0.52; *df*, 1; *p* = 0.70) (Supplementary Figures 6, 7).

However, the OR of recurrent AF or ATA after cryoballoon ablation in the elderly vs. non-elderly group was 1.12 (95% CI, 0.78–1.61; Figure 3B), with non-significant heterogeneity (Chi^2^, 0.57; *df*, 4; *I*^2^, 0%; *p* = 0.97) and non-significant publication bias according to Egger regression (*t*, 1.46; *df*, 3; *p* = 0.24) on inspection of the funnel plot (Supplementary Figure 8). In the subgroup analysis of age ≥75 and ≥80 years, the ORs (95% CI) of recurrent AF or ATA after cryoballoon ablation in the elderly vs. non-elderly groups were 1.21 (0.79–1.83) and 0.92 (0.45–1.87), respectively. There was significant publication bias according to Egger regression on inspection of the funnel plot in the subgroups of age ≥75 (*t*, 5.33; *df*, 2; *p* = 0.03) (Supplementary Figure 9).

**Figure 4:**
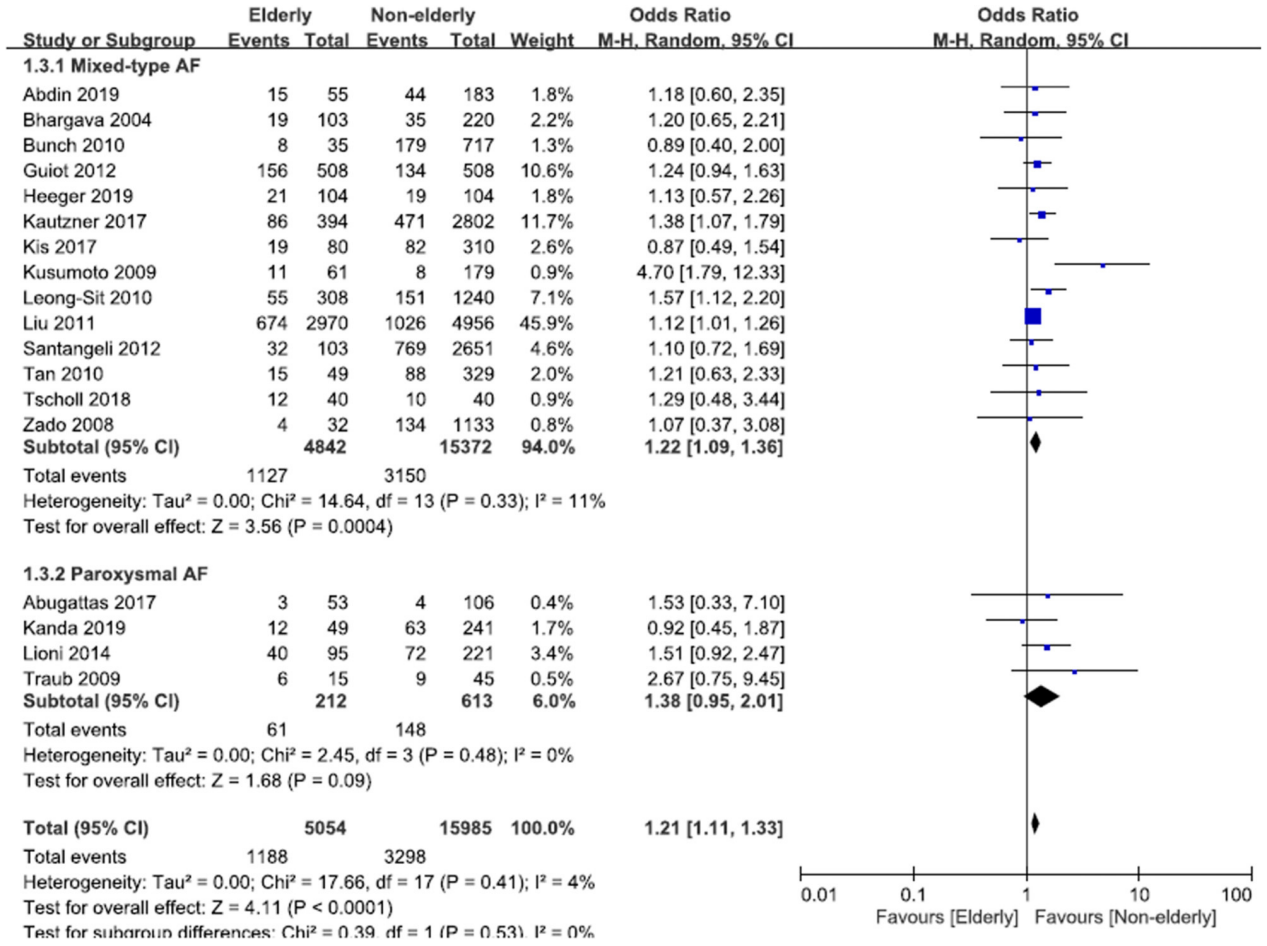
Forest plots of the OR of recurrent AF or ATA after ablation for mixed-type AF or paroxysmal AF between the elderly and non-elderly groups from 18 studies.

In the subgroups of mixed-type (paroxysmal and non-paroxysmal) AF, the OR of recurrent AF or ATA after AF ablation in the elderly vs. non-elderly groups was 1.22 (95% CI, 1.09–1.36; Figure 4), with non-significant heterogeneity (Chi^2^, 14.64; *df*, 13; *I*^2^, 11%; *p* = 0.33) and non-significant publication bias according to Egger regression (*t*, 0.96; *df*, 12; *p* = 0.35) on inspection of the funnel plot (Supplementary Figure 10). In the subgroups of paroxysmal AF, the OR of recurrent AF or ATA after ablation in the elderly vs. non-elderly groups was 1.38 (95% CI, 0.95–2.01; Figure 4), with non-significant heterogeneity (Chi^2^, 2.45; *df*, 3; *I*^2^, 0%; *p* = 0.48) and non-significant publication bias according to Egger regression (*t*, 0.42; *df*, 2; *p* = 0.71) on inspection of the funnel plot (Supplementary Figure 11).

### Pooled Odds Ratios of Complications of AF Ablation

**Figure 5:**
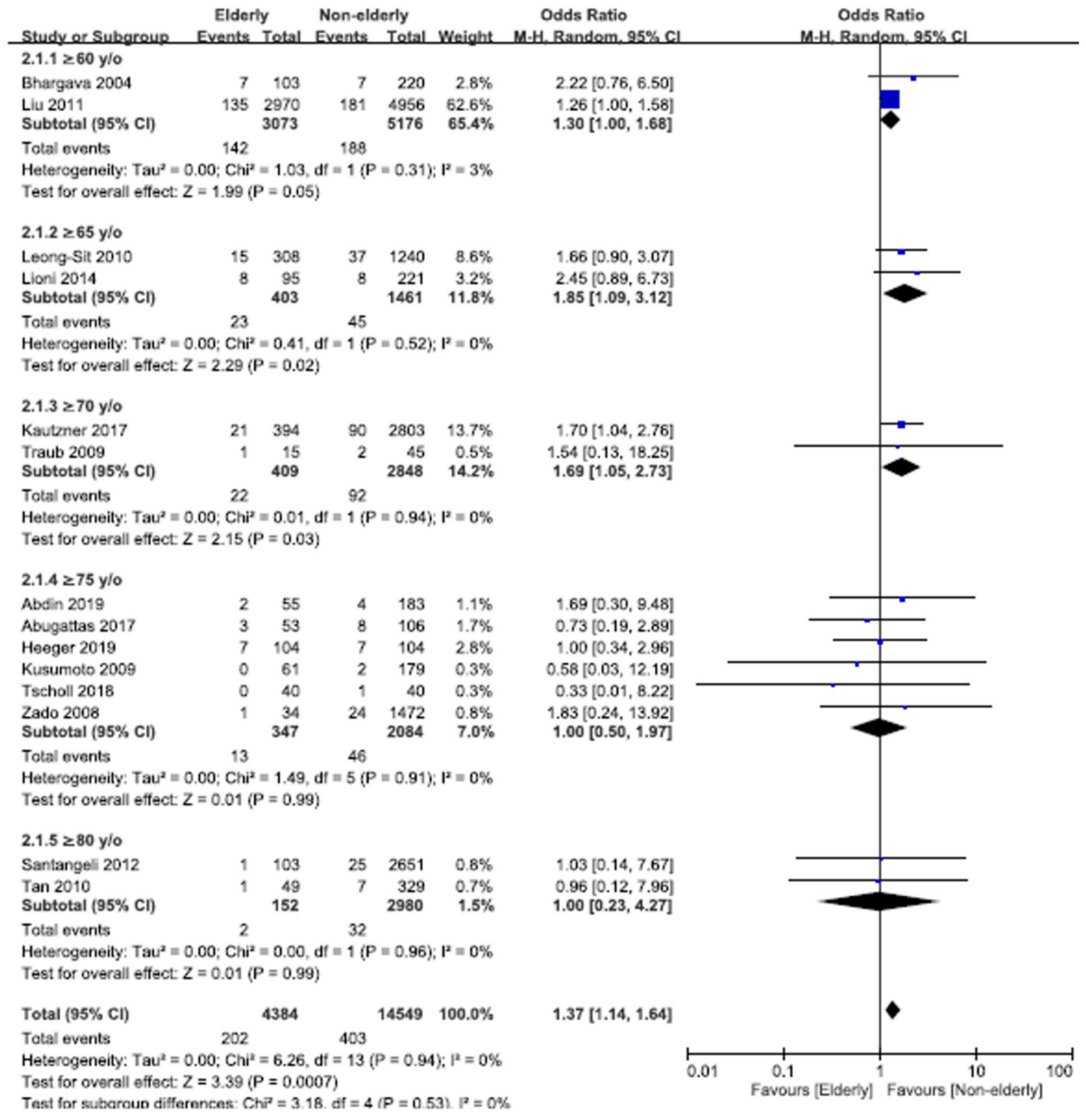
Forest plots of the overall OR of complications of AF ablation between the elderly and non-elderly groups in the entire population with different age distributions from 14 studies.

The overall OR of complications of AF ablation in the elderly vs. non-elderly group was 1.37 (95% CI, 1.14–1.64; Figure 5), with non-significant heterogeneity (Chi^2^, 6.26; *df*, 13; *I*^2^, 0%; *p* = 0.94) and non-significant publication bias according to Egger regression (*t*, 0.03; *df*, 12; *p* = 0.97) on inspection of the funnel plot (Supplementary Figure 12). In the subgroups analysis of age ≥60, ≥65, ≥70, ≥75, and ≥80 years old, the ORs (95% CI) of complications of AF ablation in the elderly vs. non-elderly groups were 1.30 (1.00–1.68), 1.85 (1.09–3.12), 1.69 (1.05–2.73), 1.00 (0.50–1.97), and 1.00 (0.23–4.27), respectively. In the subgroups of age ≥75 years old, there was non-significant heterogeneity (Chi^2^, 1.48; *df*, 5; *I*^2^, 0%; *p* = 0.92) and non-significant publication bias according to Egger regression (*t*, 0.52; *df*, 4; *p* = 0.63) on inspection of the funnel plot (Supplementary Figure 13).

**Figure 6:**
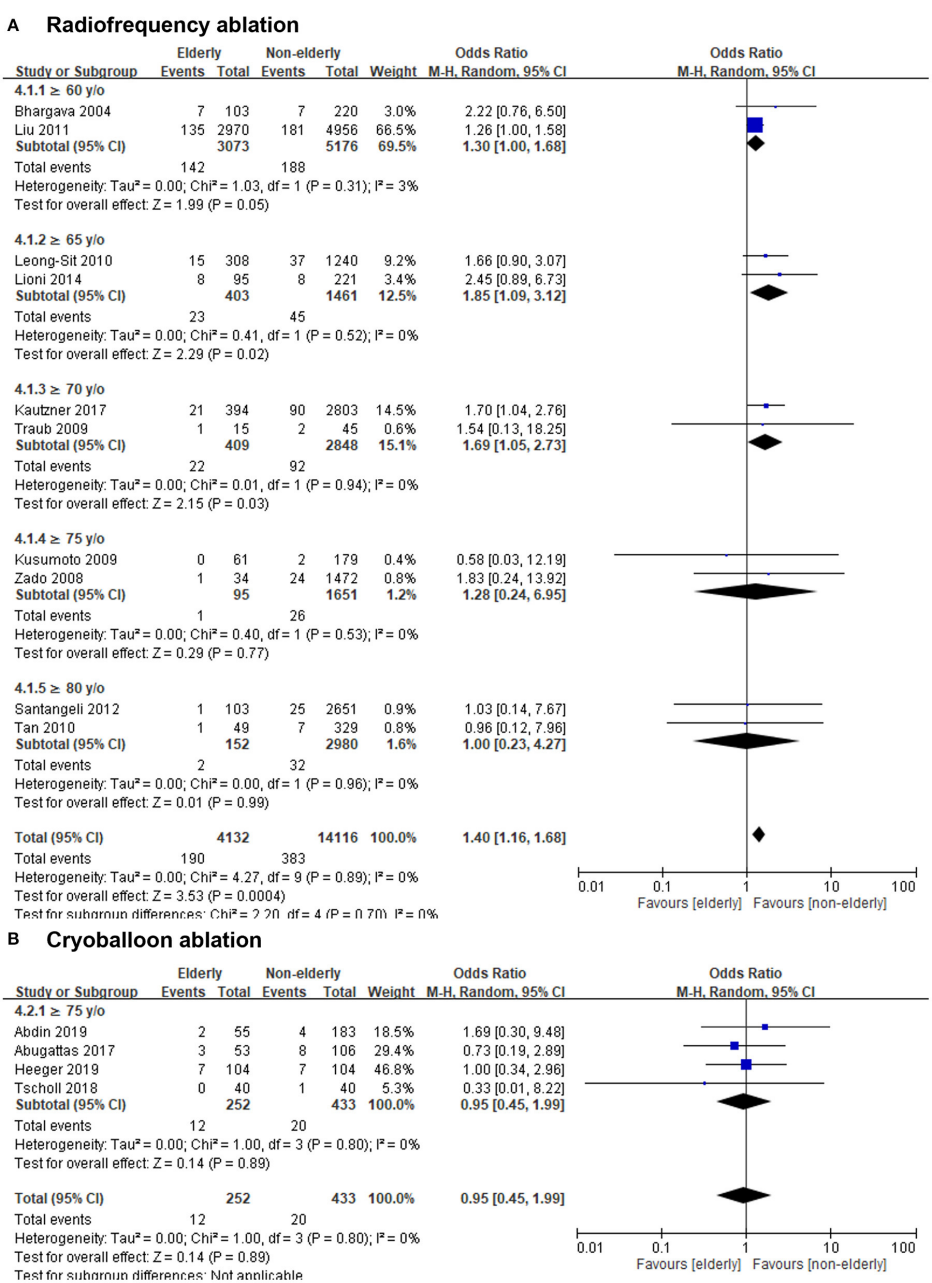
Forest plots of the OR of complications of RF ablation **(A)** or cryoballoon ablation **(B)** between the elderly and non-elderly groups in the entire population from 14 studies with different age stratification.

In terms of ablation methods, the OR of complications of RF ablation in the elderly vs. non-elderly group was 1.40 (95% CI, 1.16–1.68; Figure 6A), with non-significant heterogeneity (Chi^2^, 4.27; *df*, 9; *I*^2^, 0%; *p* = 0.89) and non-significant publication bias according to Egger regression (*t*, 0.96; *df*, 8; *p* = 0.36) on inspection of the funnel plot (Supplementary Figure 14). The OR of complications of cryoballoon ablation in the elderly vs. non-elderly group was 0.95 (95% CI, 0.45–1.99; Figure 6B), with non-significant heterogeneity (Chi^2^, 1.00; *df*, 3; *I*^2^, 0%; *p* = 0.80) and non-significant publication bias according to Egger regression (*t*, 0.62; *df*, 2; *p* = 0.60) on inspection of the funnel plot (Supplementary Figure 15).

### Standardized Mean Differences of Procedural Time and Fluoroscopic Time of AF Ablation Between the Elderly and Non-elderly Groups

**Figure 7:**
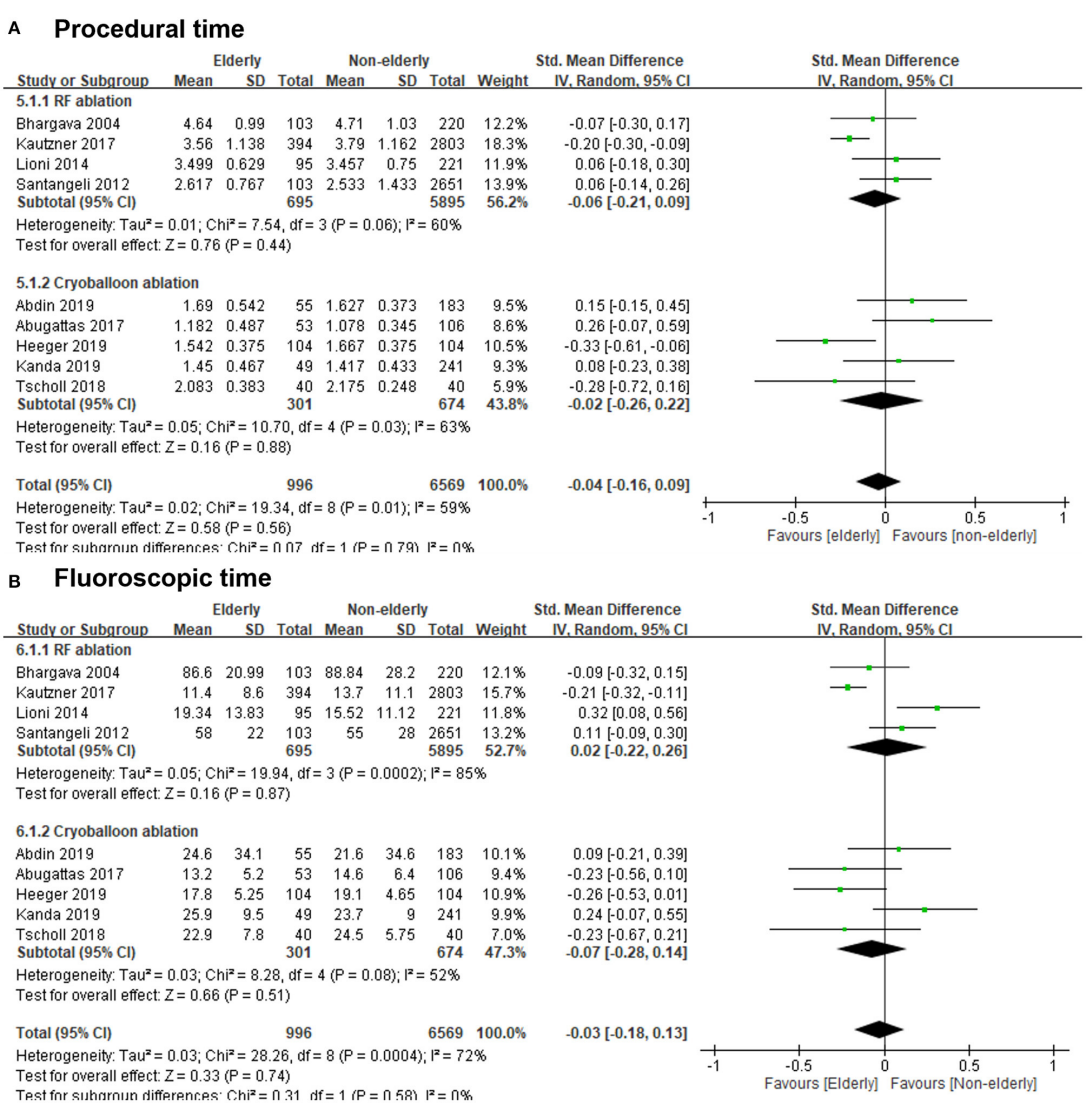
**(A)** Forest plots of the standardized mean differences (SMDs) of procedural time of RF ablation or cryoballoon ablation in the elderly vs. non-elderly group from nine studies. **(B)** Forest plots of the SMDs of fluoroscopic time of RF ablation or cryoballoon ablation in the elderly vs. non-elderly group from nine studies.

According to nine studies, the SMD of procedural time of AF ablation in the elderly vs. non-elderly group was −0.04 (95% CI, −0.16–0.09; Figure 7A), with significant heterogeneity (Chi^2^, 19.34; *df*, 8; *I*^2^, 59%; *p* = 0.01) but non-significant publication bias according to Egger regression (*t*, 1.53; *df*, 7; *p* = 0.17) on inspection of the funnel plot (Supplementary Figure 16). In terms of different ablation methods, the SMD of procedural time of RF ablation for AF in the elderly vs. non-elderly group was −0.06 (95% CI, −0.21–0.09; Figure 7A), with significant heterogeneity (Chi^2^, 7.54; *df*, 3; *I*^2^, 60%; *p* = 0.06) and non-significant publication bias according to Egger regression (*t*, 2.74; *df*, 2; *p* = 0.11) on inspection of the funnel plot (Supplementary Figure 17). The SMD of procedural time of cryoballoon ablation for AF in the elderly vs. non-elderly group was −0.02 (95% CI, −0.26–0.22; Figure 7A), with significant heterogeneity (Chi^2^, 10.70; *df*, 4; *I*^2^, 63%; *p* = 0.03) but non-significant publication bias according to Egger regression (*t*, 0.03; *df*, 3; *p* = 0.98) on inspection of the funnel plot (Supplementary Figure 18).

According to nine studies, the SMD of fluoroscopic time of AF ablation in the elderly vs. non-elderly group was −0.03 (95% CI, −0.18–0.13; Figure 7B), with significant heterogeneity (Chi^2^, 28.26; *df*, 8; *I*^2^, 72%; *p* = 0.0004) but non-significant publication bias according to Egger regression (*t*, 1.17; *df*, 7; *p* = 0.28) on inspection of the funnel plot (Supplementary Figure 19). In terms of different ablation methods, the SMD of fluoroscopic time of RF ablation for AF in the elderly vs. non-elderly group was 0.02 (95% CI, −0.22–0.26; Figure 7B), with significant heterogeneity (Chi^2^, 19.94; *df*, 3; *I*^2^, 85%; *p* = 0.0002) but non-significant publication bias according to Egger regression (*t*, 2.24; *df*, 2; *p* = 0.15) on inspection of the funnel plot (Supplementary Figure 20). The SMD of fluoroscopic time of cryoballoon ablation for AF in the elderly group vs. non-elderly group was −0.07 (95% CI, −0.28–0.14; Figure 7B), with significant heterogeneity (Chi^2^, 8.28; *df*, 4; *I*^2^, 52%; *p* = 0.08) but non-significant publication bias according to Egger regression (*t*, 0.24; *df*, 3; *p* = 0.82) on inspection of the funnel plot (Supplementary Figure 21).

## Discussion

This meta-analysis study showed that elderly patients with AF had a significantly higher incidence of recurrent AF or ATA after AF ablation compared to non-elderly patients with AF (<60 years old), except in patients ≥75 years old. Compared to non-elderly patients with AF, elderly patients with AF had a significantly higher incidence of recurrent AF or ATA after AF ablation for mixed-type (paroxysmal and non-paroxysmal) AF. However, there was no difference in terms of recurrent AF or ATA after AF ablation for paroxysmal AF between elderly and non-elderly patients with AF. The elderly patients with AF had a significantly higher incidence of complication of AF ablation compared to non-elderly patients with AF, except in patients ≥75 years old. There was no difference in the procedure time and fluoroscopic time between elderly and non-elderly patients AF. The elderly group had significantly higher prevalence of diabetes mellitus, hypertension, heart failure, coronary artery disease, and paroxysmal AF compared to the non-elderly group. Except for age, more comorbidities also influenced the incidence of recurrent AF or ATA after AF ablation.

AF is a progressive and an important disease in the elderly population. In addition, age has a great impact in the development of AF and imposes significant limitations in the treatment of AF because of the higher prevalence of conduction abnormalities that limits the use of pharmacological rate or rhythm control strategies (29). AF ablation is effective in achieving and maintaining sinus rhythm, and is associated with lower mortality, improved quality of life, and a lower risk of progression to permanent AF (29, 30). Ablation is an effective strategy in treating symptomatic AF in selected elderly patients as a stand-alone therapy or as hybrid therapy with anti-arrhythmic medication, and is associated with decreased healthcare resource utilization in all age groups (19, 20). The current guidelines recommended ablation strategy for patients with symptomatic AF associated with heart failure and not specific comment of ablation strategy for the elderly patients (7, 8). Traditionally, the definition of elderly was aged 60 or 65 years and over (31). Recently, some researchers redefined elderly as age ≥75 years old (32).

Many papers reported similar efficacy of AF ablation (11, 12, 14–16, 18–28), and similar safety in elderly patients (15–28). Leong-Sit et al. reported a relatively lower complication rate and higher possibilities in freedom from AF off anti-arrhythmic medication in the younger population (<45 years old) (13). This population (<45 years old) presented significantly smaller left atrial size and zero point of CHADS2 score, indicating less structural and electrical remodeling of the left atrium. Kautzner et al. also reported a significantly higher prevalence of good arrhythmia control without anti-arrhythmic medication in the younger population (<70 years old vs. >70 years old; 58.2 vs. 44.2%; *p* < 0.001) (18). Guiot et al. also reported that age >75 years was the only predictor of cerebrovascular events after AF ablation in patients ≥65 years old (14). However, all studies were observational studies, and most had limited patient numbers. Of note, in this study, we found that elderly AF patients with age ≥75 years old had similar incidence of recurrent AF or ATA and complication after AF ablation compared to non-elderly patients with AF (<60 years old). However, elderly patients with AF (60–74 years old) had a significantly higher incidence of recurrent AF or ATA and complication after AF ablation compared to non-elderly patients with AF (<60 years old). The mechanisms responsible for discrepancy remain unexplored. Most aged patients had structural and electrical remodeling in left atrium due to longer AF duration and had more comorbidities (5, 12, 15). After ablation, the degree of reverse electrical and structural remodeling of the left atrium may be influenced by longer AF duration and more comorbidities (33). In addition, a higher prevalence of AF or ATA originating from the non-pulmonary vein triggers in the aged population could contribute to the recurrence of AF or ATA by ablation strategy with pulmonary vein isolation alone. However, more ablation to include non-pulmonary vein triggers may increase the risk of complications.

## Limitations

This study has several limitations. First, all studies were observational cohort studies and not all studies provided detailed information about the AF ablation procedure. Second, the age distribution in each study was not the same. However, a total of 21,039 participants were enrolled from 18 studies. The present study provides important findings on the outcomes of AF ablation in the elderly population with AF. However, large and randomized studies are warranted to validate these findings.

## Conclusion

The elderly patients with AF had significantly higher incidences of recurrent AF or ATA and complications after ablation for non-paroxysmal AF compared to the non-elderly patients with AF (<60 years old). However, the efficacy and safety of AF ablation in AF patients ≥75 years old were similar to those of non-elderly patients with AF.

## Data Availability Statement

The raw data supporting the conclusions of this article will be made available by the authors, without undue reservation.

## Author Contributions

W-CL and P-JW reviewed the articles and wrote the manuscript. H-YF and H-CC prepared figures. P-YL and M-CC did the final revision. All authors reviewed the manuscript.

## Conflict of Interest

The authors declare that the research was conducted in the absence of any commercial or financial relationships that could be construed as a potential conflict of interest.

## Publisher's Note

All claims expressed in this article are solely those of the authors and do not necessarily represent those of their affiliated organizations, or those of the publisher, the editors and the reviewers. Any product that may be evaluated in this article, or claim that may be made by its manufacturer, is not guaranteed or endorsed by the publisher.

## References

[1] Aronow WS Banach M. Atrial fibrillation: the new epidemic of the ageing world. J Atr Fibrillation. (2009) 1:154. 10.4022/jafib.v1i6.530 28496617 PMC5398780

[2] Béjot Y Bailly H Graber M Garnier L Laville A Dubourget L. Impact of the ageing population on the burden of stroke: the dijon stroke registry. Neuroepidemiology. (2019) 52:78–85. 10.1159/000492820 30602168

[3] Nantsupawat T Nugent K Phrommintikul A. Atrial fibrillation in the elderly. Drugs Aging. (2013) 30:593–601. 10.1007/s40266-013-0094-8 23709402

[4] Lee HC Tl Huang K Shen WK. Use of antiarrhythmic drugs in elderly patients. J Geriatr Cardiol. (2011) 8:184–94. 10.3724/SP.J.1263.2011.00184 22783304 PMC3390066

[5] Dun W Boyden PA. Aged atria: electrical remodeling conducive to atrial fibrillation. J Interv Card Electrophysiol. (2009) 25:9–18. 10.1007/s10840-008-9358-3 19280327 PMC4332532

[6] Lin YK Chen YA Lee TI Chen YC Chen SA Chen YJ. Aging modulates the substrate and triggers remodeling in atrial fibrillation. Circ J. (2018) 82:1237–44. 10.1253/circj.CJ-17-0242 28904308

[7] Calkins H Hindricks G Cappato R Kim YH Saad EB Aguinaga L. 2017 HRS/EHRA/ECAS/APHRS/SOLAECE expert consensus statement on catheter and surgical ablation of atrial fibrillation. Europace. (2018) 20:e1–160. 10.1093/europace/eux274 29016840 PMC5834122

[8] Hindricks G Potpara T Dagres N Arbelo E Bax JJ Blomström-Lundqvist C. 2020 ESC Guidelines for the diagnosis and management of atrial fibrillation developed in collaboration with the European Association for Cardio-Thoracic Surgery (EACTS). Eur Heart J. (2021) 42:373–498. 10.1016/j.rec.2021.03.009 32860505

[9] Marrouche NF Brachmann J Andresen D Siebels J Boersma L Jordaens L. Catheter ablation for atrial fibrillation with heart failure. N Engl J Med. (2018) 378:417–27. 10.1056/NEJMoa1707855 29385358

[10] Danielsen R Thorgeirsson G Einarsson H Ólafsson Ö Aspelund T Harris TB Launer L. Prevalence of heart failure in the elderly and future projections: the AGES-Reykjavík study. Scand Cardiovasc J. (2017) 51:183–9. 10.1080/14017431.2017.1311023 28366010 PMC5681737

[11] Bhargava M Marrouche NF Martin DO Schweikert RA Saliba W Saad EB. Impact of age on the outcome of pulmonary vein isolation for atrial fibrillation using circular mapping technique and cooled-tip ablation catheter. J Cardiovasc Electrophysiol. (2004) 15:8–13. 10.1046/j.1540-8167.2004.03266.x 15028066

[12] Liu Y Huang H Huang C Zhang S Ma C Liu X. Catheter ablation of atrial fibrillation in Chinese elderly patients. Int J Cardiol. (2011) 152:266–7. 10.1016/j.ijcard.2011.07.104 21864920

[13] Leong-Sit P Zado E Callans DJ Garcia F Lin D Dixit S. Efficacy and risk of atrial fibrillation ablation before 45 years of age. Circ Arrhythm Electrophysiol. (2010) 3:452–7. 10.1161/CIRCEP.110.938860 20858861

[14] Guiot A Jongnarangsin K Chugh A Suwanagool A Latchamsetty R Myles JD. Anticoagulant therapy and risk of cerebrovascular events after catheter ablation of atrial fibrillation in the elderly. J Cardiovasc Electrophysiol. (2012) 23:36–43. 10.1111/j.1540-8167.2011.02141.x 21806701

[15] Lioni L Letsas KP Efremidis M Vlachos K Giannopoulos G Kareliotis V. Catheter ablation of atrial fibrillation in the elderly. J Geriatr Cardiol. (2014) 11:291–5. 10.11909/j.issn.1671-5411.2014.04.003 25593577 PMC4294145

[16] Kis Z Noten AM Martirosyan M Hendriks AA Bhagwandien R Szili-Torok T. Comparison of long-term outcome between patients aged <65 years vs. ≥ 65 years after atrial fibrillation ablation. J Geriatr Cardiol. (2017) 14:569–74. 10.11909/j.issn.1671-5411.2017.09.004 29056955 PMC5641644

[17] Traub D Daubert JP McNitt S Zareba W Hall B. Catheter ablation of atrial fibrillation in the elderly: where do we stand? Cardiol J. (2009) 16:113–20. 19387957

[18] Kautzner J Peichl P Sramko M Cihak R Aldhoon B Wichterle D. Catheter ablation of atrial fibrillation in elderly population. J Geriatr Cardiol. (2017) 14:563–8. 10.11909/j.issn.1671-5411.2017.09.008 29144514 PMC5641643

[19] Zado E Callans DJ Riley M Hutchinson M Garcia F Bala R. Long-term clinical efficacy and risk of catheter ablation for atrial fibrillation in the elderly. J Cardiovasc Electrophysiol. (2008) 19:621–6. 10.1111/j.1540-8167.2008.01183.x 18462325

[20] Kusumoto F Prussak K Wiesinger M Pullen T Lynady C. Radiofrequency catheter ablation of atrial fibrillation in older patients: outcomes and complications. J Interv Card Electrophysiol. (2009) 25:31–5. 10.1007/s10840-008-9346-7 19148720

[21] Abugattas JP Iacopino S Moran D De Regibus V Takarada K Mugnai G. Efficacy and safety of the second generation cryoballoon ablation for the treatment of paroxysmal atrial fibrillation in patients over 75 years: a comparison with a younger cohort. Europace. (2017) 19:1798–803. 10.1093/europace/eux023 28402529

[22] Tscholl V Lin T Lsharaf AK Bellmann B Nagel P Lenz K. Cryoballoon ablation in the elderly: one year outcome and safety of the second-generation 28mm cryoballoon in patients over 75 years old. Europace. (2018) 20:772–7. 10.1093/europace/eux128 29741689

[23] Abdin A Yalin K Lyan E Sawan N Liosis S Meyer-Saraei R. Safety and efficacy of cryoballoon ablation for the treatment of atrial fibrillation in elderly patients. Clin Res Cardiol. (2019) 108:167–74. 10.1007/s00392-018-1336-x 30187178

[24] Heeger CH Bellmann B Fink T Bohnen JE Wissner E Wohlmuth P. Efficacy and safety of cryoballoon ablation in the elderly: A multicenter study. Int J Cardiol. (2019) 278:108–13. 10.1016/j.ijcard.2018.09.090 30287056

[25] Bunch TJ Weiss JP Crandall BG May HT Bair TL Osborn JS. Long-term clinical efficacy and risk of catheter ablation for atrial fibrillation in octogenarians. Pacing Clin Electrophysiol. (2010) 33:146–52. 10.1111/j.1540-8159.2009.02604.x 19889181

[26] Tan HW Wang XH Shi HF Yang GS Zhou L Gu JN. Efficacy, safety and outcome of catheter ablation for atrial fibrillation in octogenarians. Int J Cardiol. (2010) 145:147–8. 10.1016/j.ijcard.2009.06.055 19616862

[27] Santangeli P Di Biase L Mohanty P Burkhardt JD Horton R Bai R. Catheter ablation of atrial fibrillation in octogenarians: safety and outcomes. J Cardiovasc Electrophysiol. (2012) 23:687–93. 10.1111/j.1540-8167.2012.02293.x 22494628

[28] Kanda T Masuda M Kurata N Asai M Iida O Okamoto S. Efficacy and safety of the cryoballoon-based atrial fibrillation ablation in patients aged ≥80 years. J Cardiovasc Electrophysiol. (2019) 30:2242–7. 10.1111/jce.14166 31507014

[29] Rojas F Valderrábano M. Effect of age on outcomes of catheter ablation of atrial fibrillation. J Atr Fibrillation. (2013) 6:886. 10.4022/jafib.886 28496863 PMC5153072

[30] Pappone C Radinovic A Manguso F Vicedomini G Ciconte G Sacchi S. Atrial fibrillation progression and management: a 5-year prospective follow-up study. Heart Rhythm. (2008) 5:1501–7. 10.1016/j.hrthm.2008.08.011 18842464

[31] Orimo H. [Reviewing the definition of elderly]. Nihon Ronen Igakkai Zasshi. (2006) 43:27–34. 10.3143/geriatrics.43.27 16521795

[32] Ouchi Y Rakugi H Arai H Akishita M Ito H Toba K. Redefining the elderly as aged 75 years and older: Proposal from the Joint Committee of Japan Gerontological Society and the Japan Geriatrics Society. Geriatr Gerontol Int. (2017) 17:1045–7. 10.1111/ggi.13118 28670849

[33] Chung MK Eckhardt LL Chen LY Ahmed HM Gopinathannair R Joglar JA. Lifestyle and risk factor modification for reduction of atrial fibrillation: a scientific statement from the American heart association. Circulation. (2020) 141:e750–72. 10.1161/CIR.0000000000000748 32148086

