# Supplementary material for: Efficacy and Safety of Ablation for Symptomatic Atrial Fibrillation in Elderly Patients: A Meta-Analysis
Source: Front Cardiovasc Med. 2021 Sep 20;8:734204. doi: 10.3389/fcvm.2021.734204 (PMC8489560; doi:10.3389/fcvm.2021.734204)
Supplement: Supplementary file 1 [file Data_Sheet_1.PDF]

Supplemental Table 1.

Risk of Bias Assessment by the Risk Of Bias In Non-Randomized Studies Of Interventions (ROBINS-I) tool

Studies	Confounding	Selection	Measurement of exposure	Departures from exposure	Missing data	Measurement of outcomes	Reported results	Power
Bhargava M (2004) [11]								
Liu Y (2011) [12]								
Leong-Sit P (2010) [13]								
Guiot A (2012) [14]								
Lioni L (2014) [15]								
Kis Z (2017) [16]								
Traub D (2009) [17]								
Kautzner J (2017) [18]								
Zado E (2008) [19]								
Kusumoto F (2009) [20]								
Abugattas JP (2017) [21]								
Tscholl V (2018) [22]								
Abdin A (2019) [23]								
Heeger CH (2019) [24]								
Bunch TJ (2010) [25]								

Tan HW (2010) [26]								
Santangeli P (2012) [27]								
Kanda T (2019) [28]								

Low	Moderate	Serious	Critical
