# Supplementary material for: Efficacy and Safety of Ablation for Symptomatic Atrial Fibrillation in Elderly Patients: A Meta-Analysis
Source: Front Cardiovasc Med. 2021 Sep 20;8:734204. doi: 10.3389/fcvm.2021.734204 (PMC8489560; doi:10.3389/fcvm.2021.734204)
Supplement: Supplementary file 2 [file Data_Sheet_2.PDF]

Supplemental Figure Legends

Figure 1.

The funnel plot showed non-significant publication bias according to Egger regression (t, 1.33; df, 16; p = 0.20).

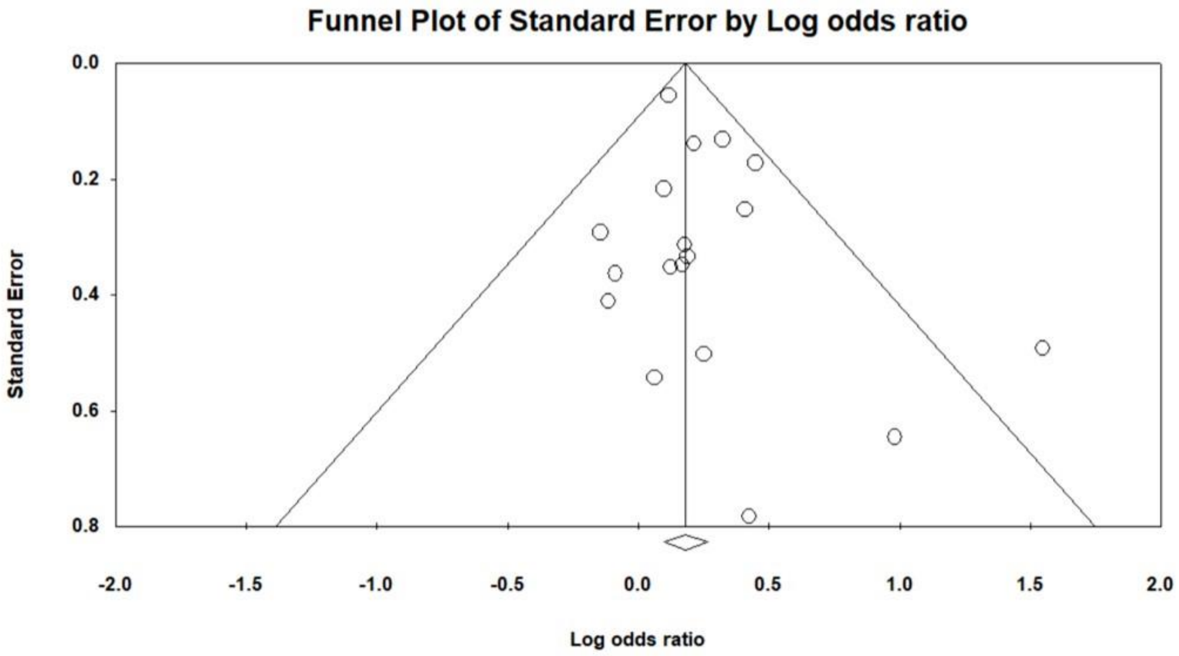

Figure 2.

The funnel plot showed non-significant publication bias according to Egger regression (t, 0.44; df, 2; p = 0.71).

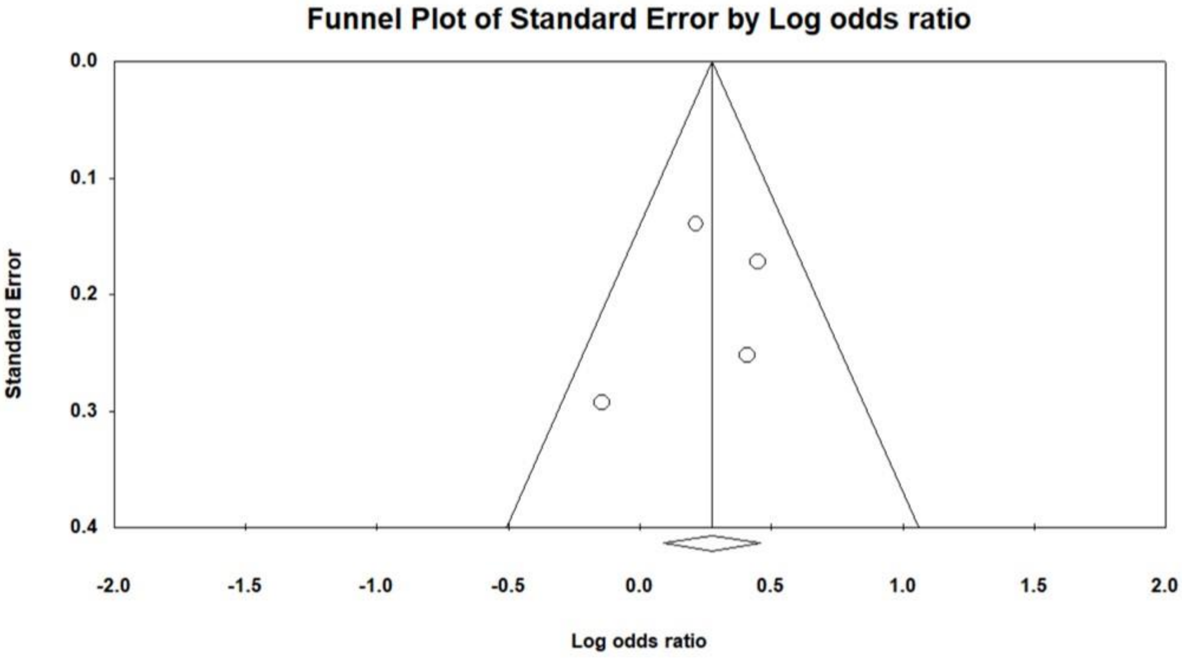

Figure 3.

The funnel plot showed non-significant publication bias according to Egger regression (*t*, 0.60; *df*, 4; *p* = 0.58).

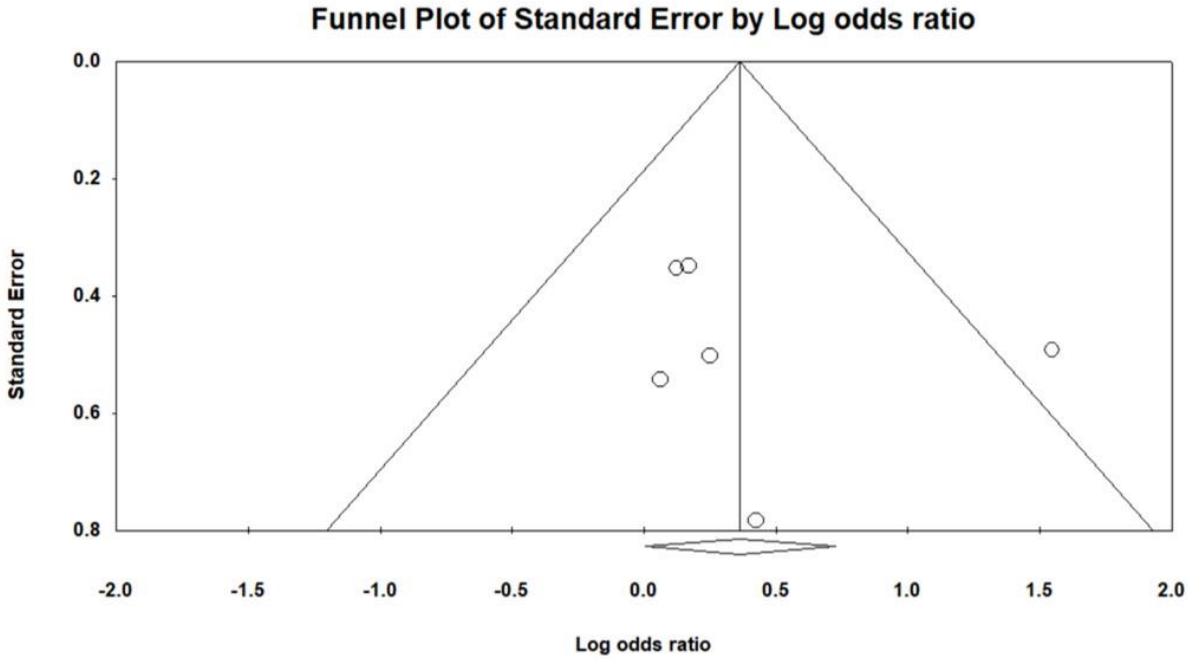

Figure 4.

The funnel plot showed non-significant publication bias according to Egger regression (*t*, 1.02; *df*, 2; *p* = 0.42).

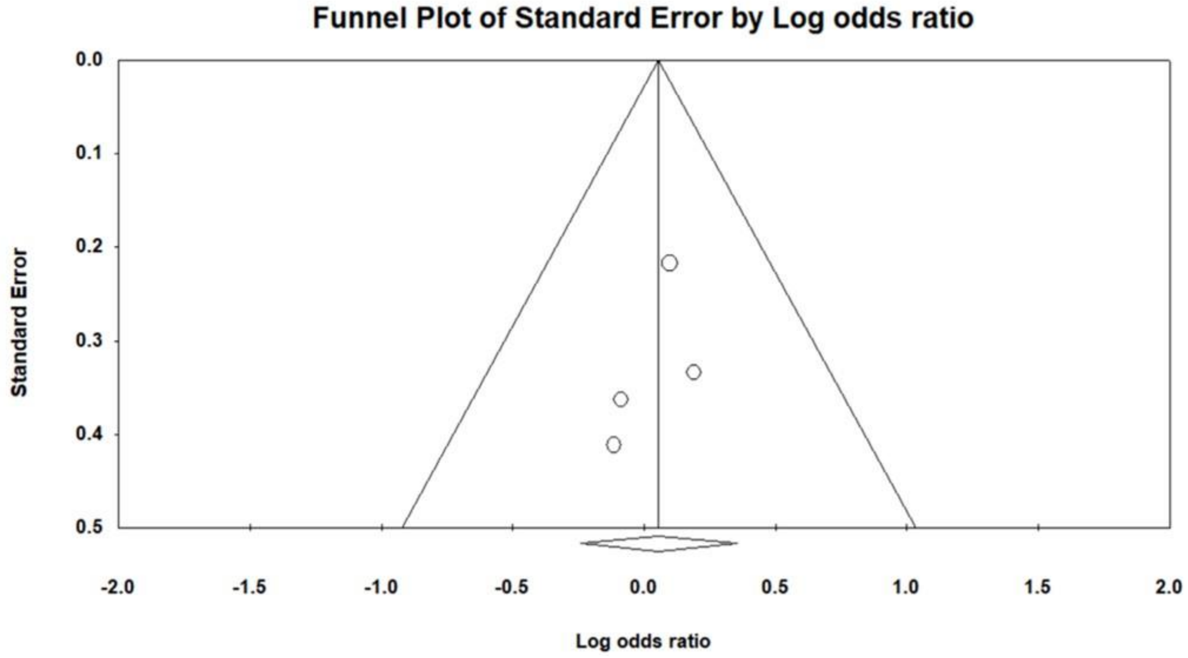

Figure 5.

The funnel plot showed significant publication bias according to Egger regression (*t*, 1.96; *df*, 10; *p* = 0.08).

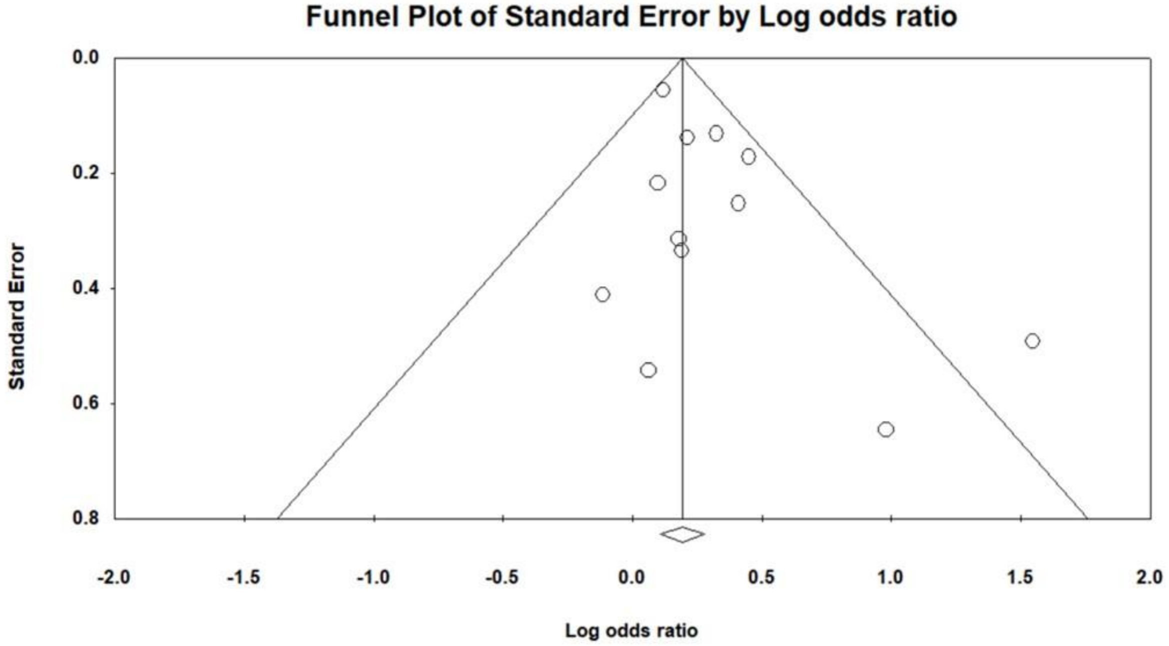

Figure 6.

The funnel plot showed non-significant publication bias according to Egger regression (*t*, 0.88; *df*, 1; *p* = 0.54).

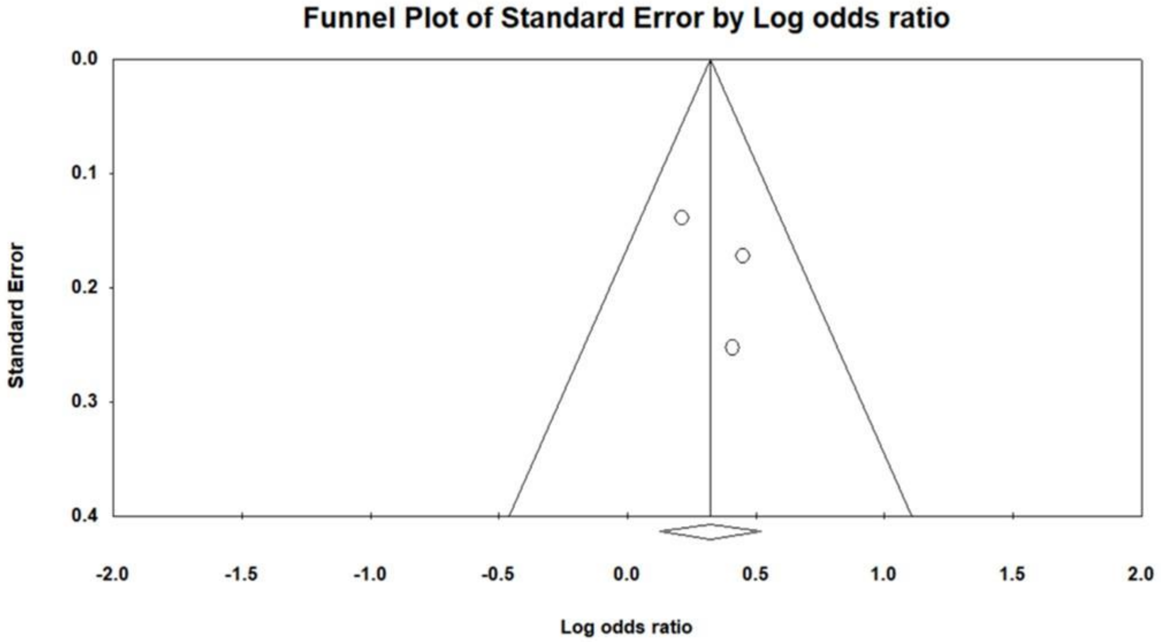

Figure 7.

The funnel plot showed non-significant publication bias according to Egger

regression (*t*, 0.52; *df*, 1; *p* = 0.70).

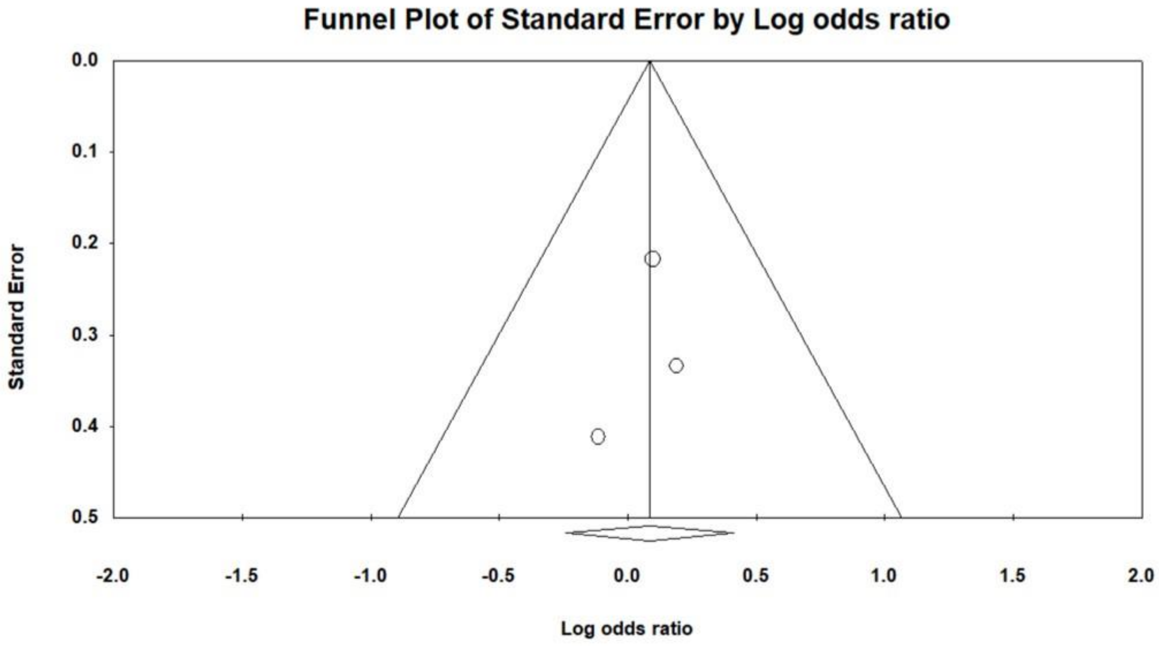

Figure 8.

The funnel plot showed non-significant publication bias according to Egger regression (*t*, 1.46; *df*, 3; *p* = 0.24).

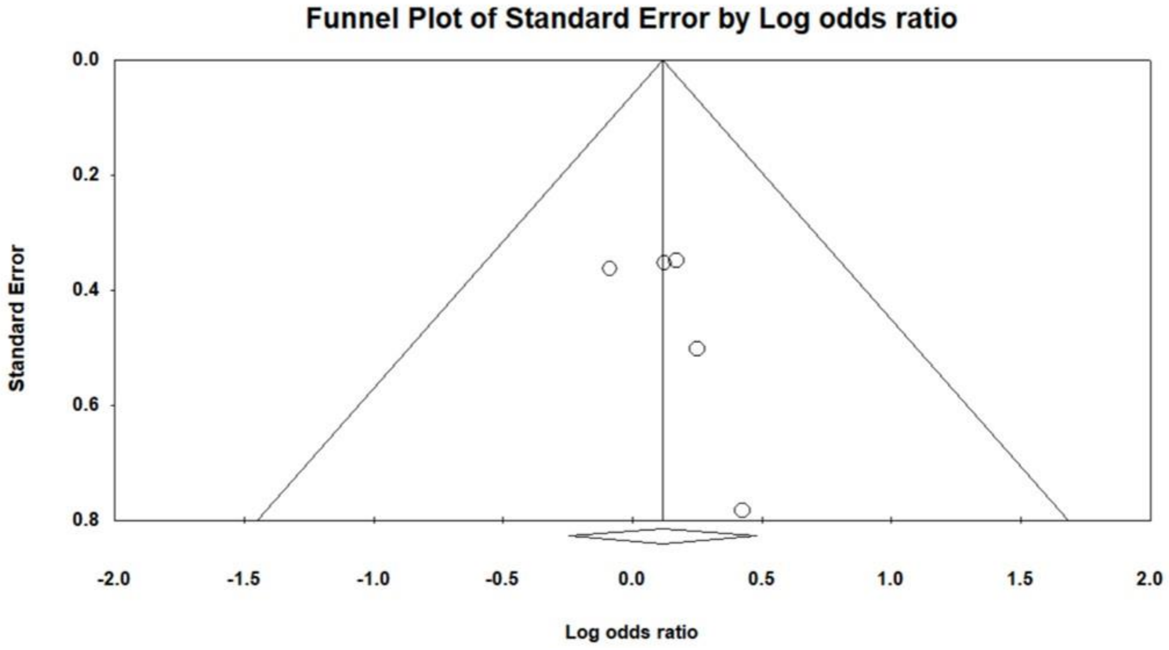

Figure 9.

The funnel plot showed significant publication bias according to Egger regression (*t*, 5.33; *df*, 2; *p* = 0.03).

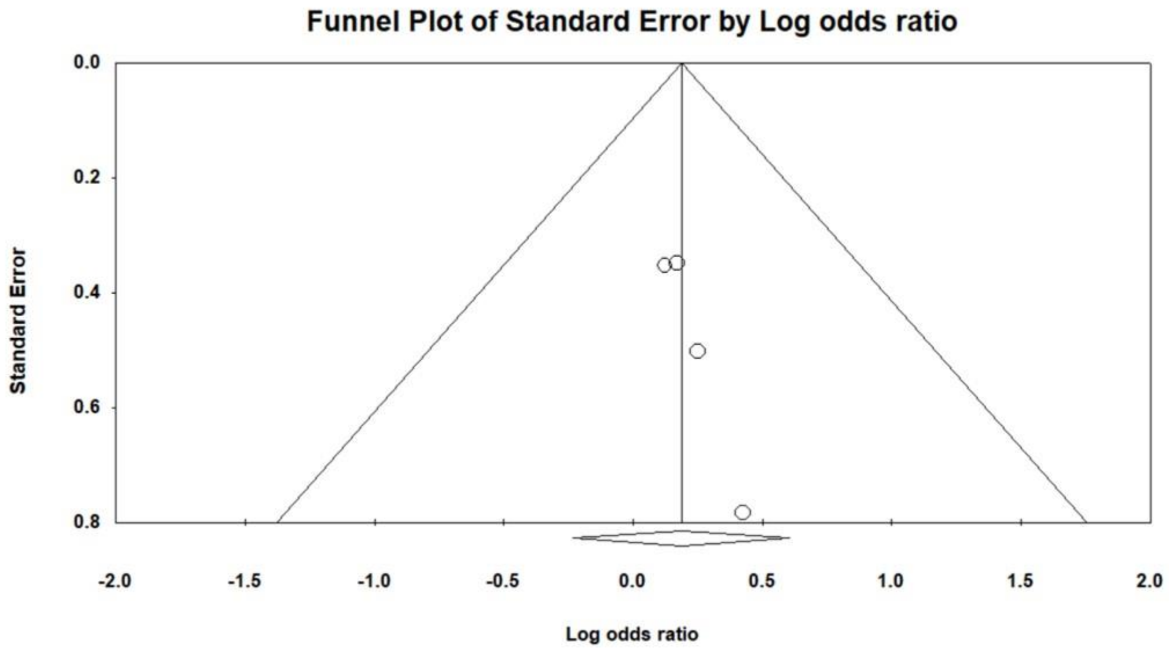

Figure 10.

The funnel plot showed non-significant publication bias according to Egger regression (*t*, 0.96; *df*, 12; *p* = 0.35).

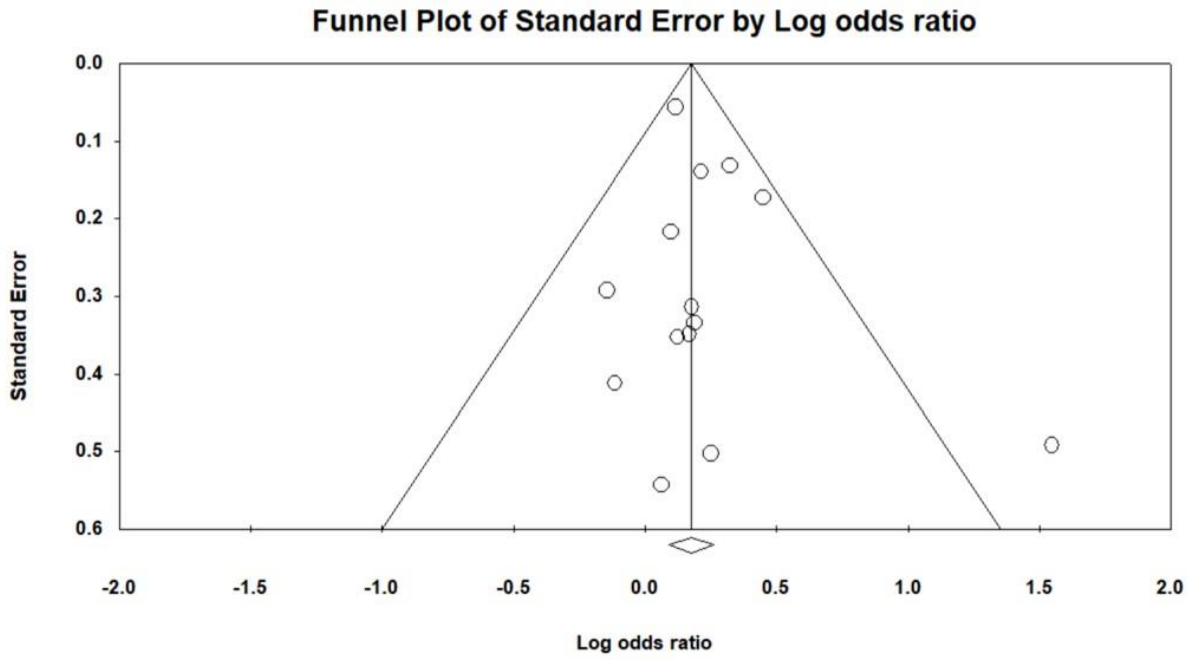

Figure 11.

The funnel plot showed non-significant publication bias according to Egger regression (*t*, 0.42; *df*, 2; *p* = 0.71).

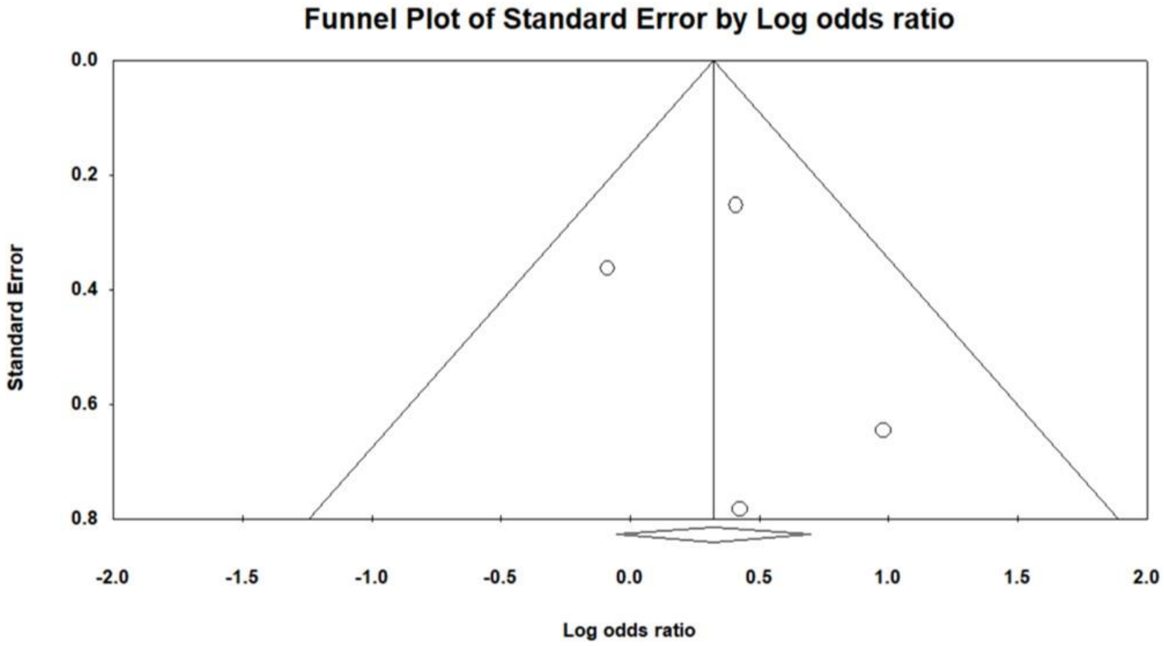

Figure 12.

The funnel plot showed non-significant publication bias according to Egger regression (*t*, 0.03; *df*, 12; *p* = 0.97).

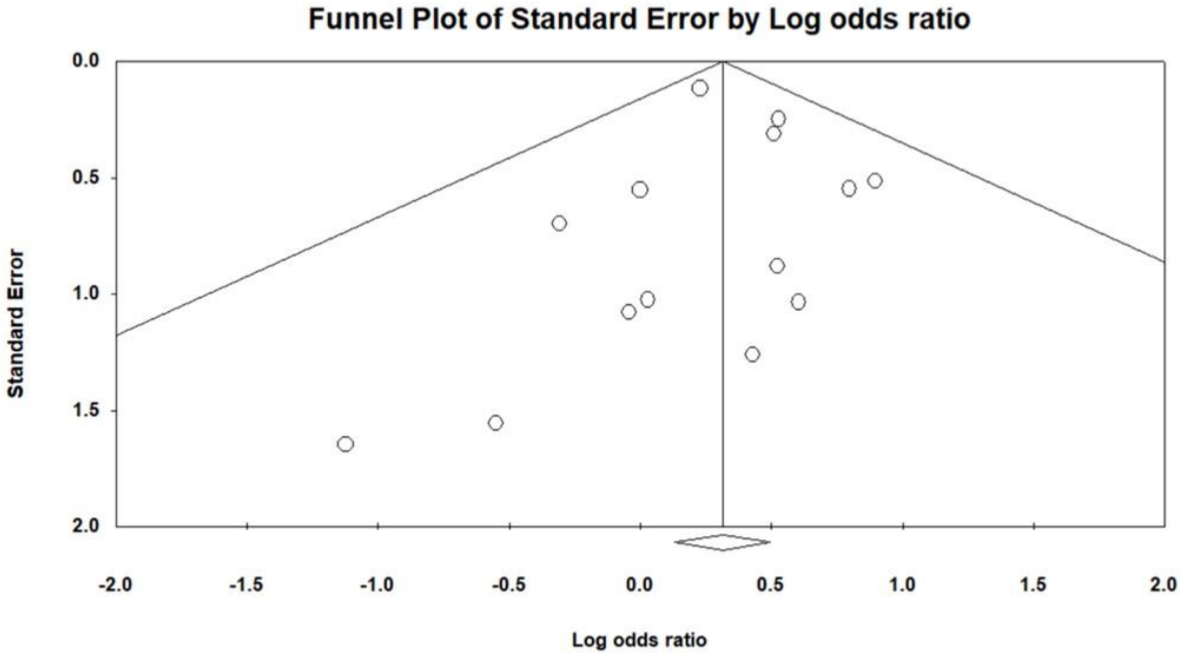

Figure 13.

The funnel plot showed non-significant publication bias according to Egger regression (*t*, 0.52; *df*, 4; *p* = 0.63).

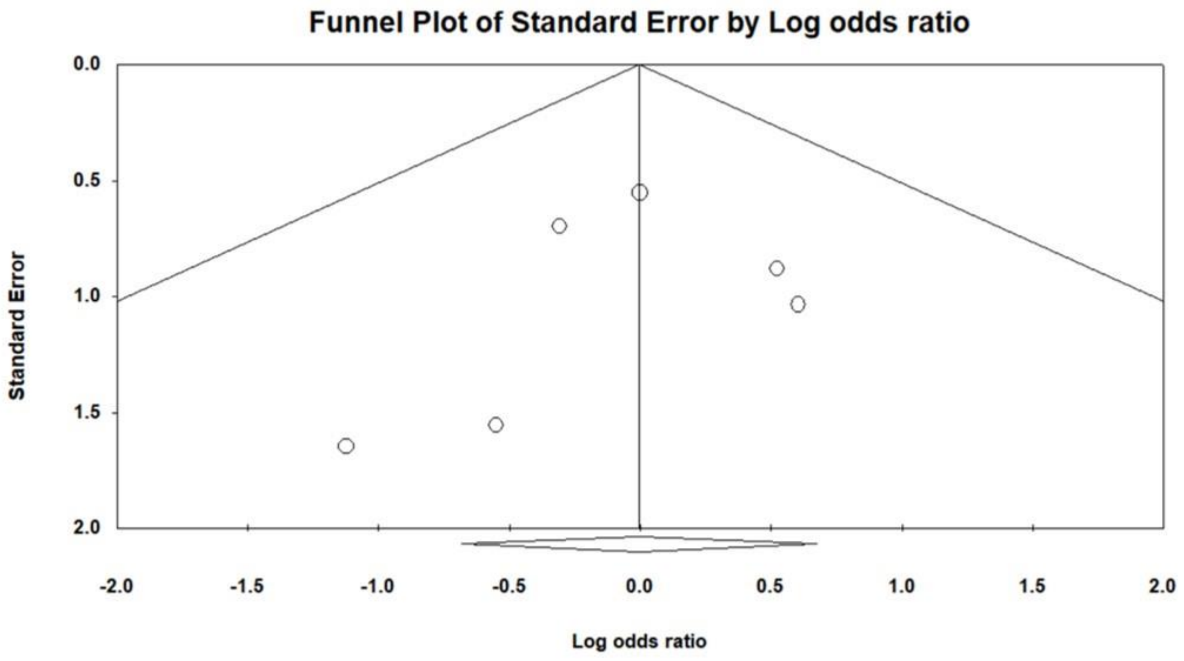

Figure 14.

The funnel plot showed non-significant publication bias according to Egger regression (*t*, 0.96; *df*, 8; $p = 0.36$).

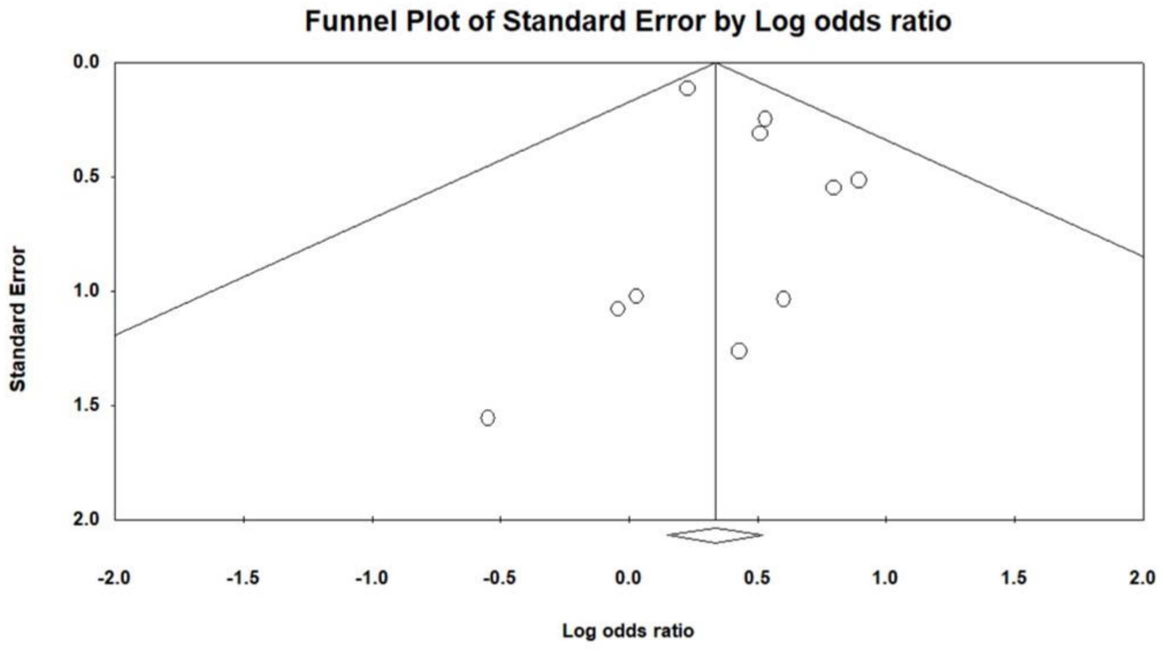

Figure 15.

The funnel plot showed non-significant publication bias according to Egger regression (*t*, 0.62; *df*, 2; $p = 0.60$).

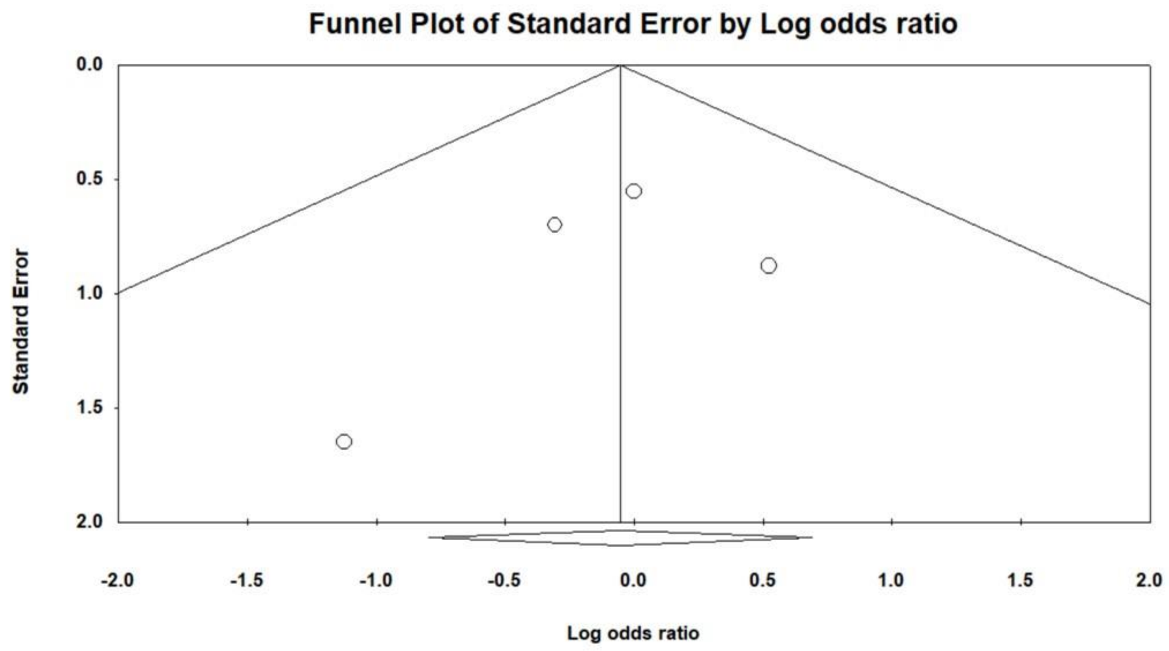

Figure 16.

The funnel plot showed non-significant publication bias according to Egger regression (*t*, 1.53; *df*, 7; *p* = 0.17).

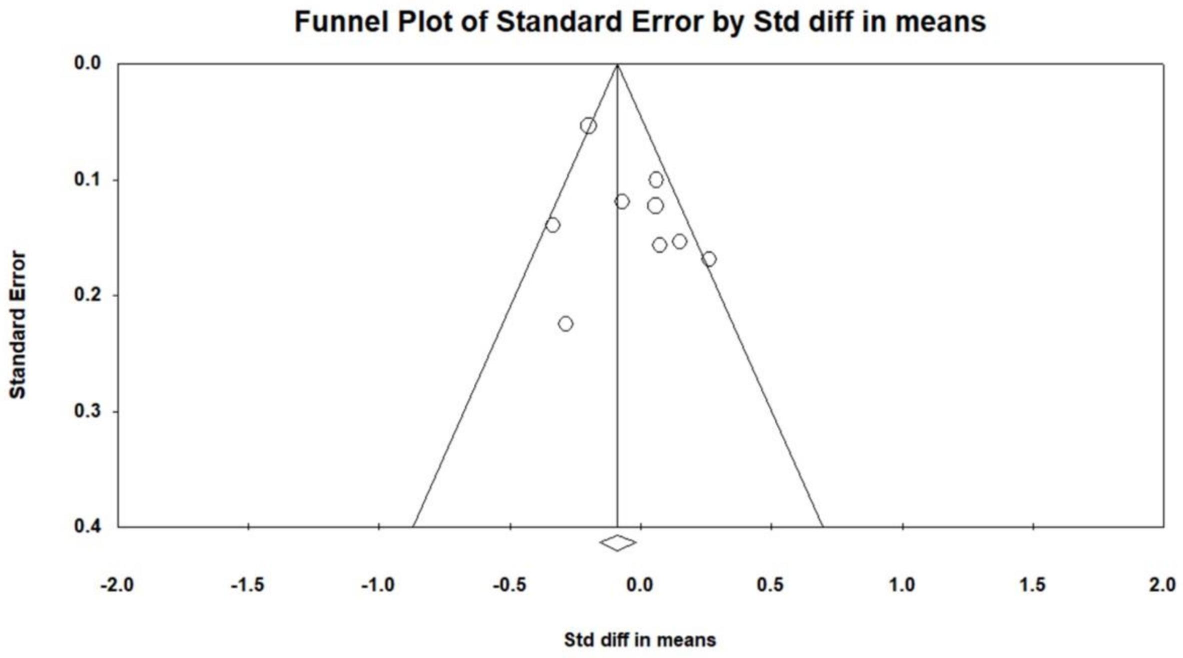

Figure 17.

The funnel plot showed non-significant publication bias according to Egger regression (*t*, 2.74; *df*, 2; *p* = 0.11).

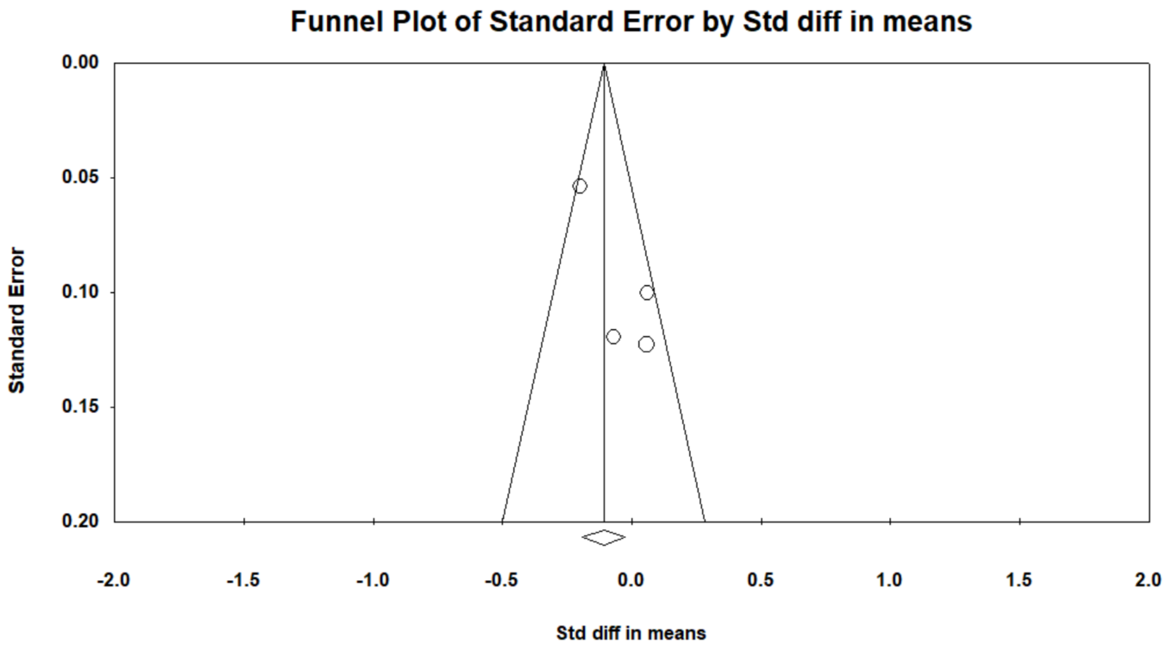

Figure 18.

The funnel plot showed non-significant publication bias according to Egger regression (*t*, 0.03; *df*, 3; *p* = 0.98).

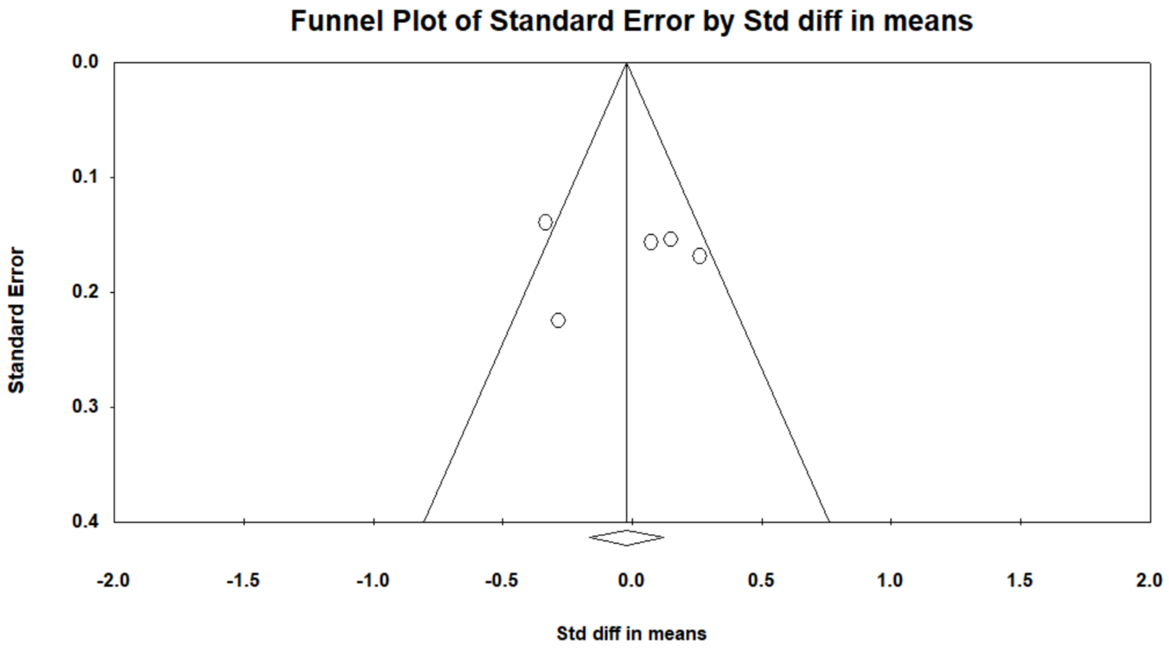

Figure 19.

The funnel plot showed non-significant publication bias according to Egger regression (*t*, 1.17; *df*, 7; *p* = 0.28).

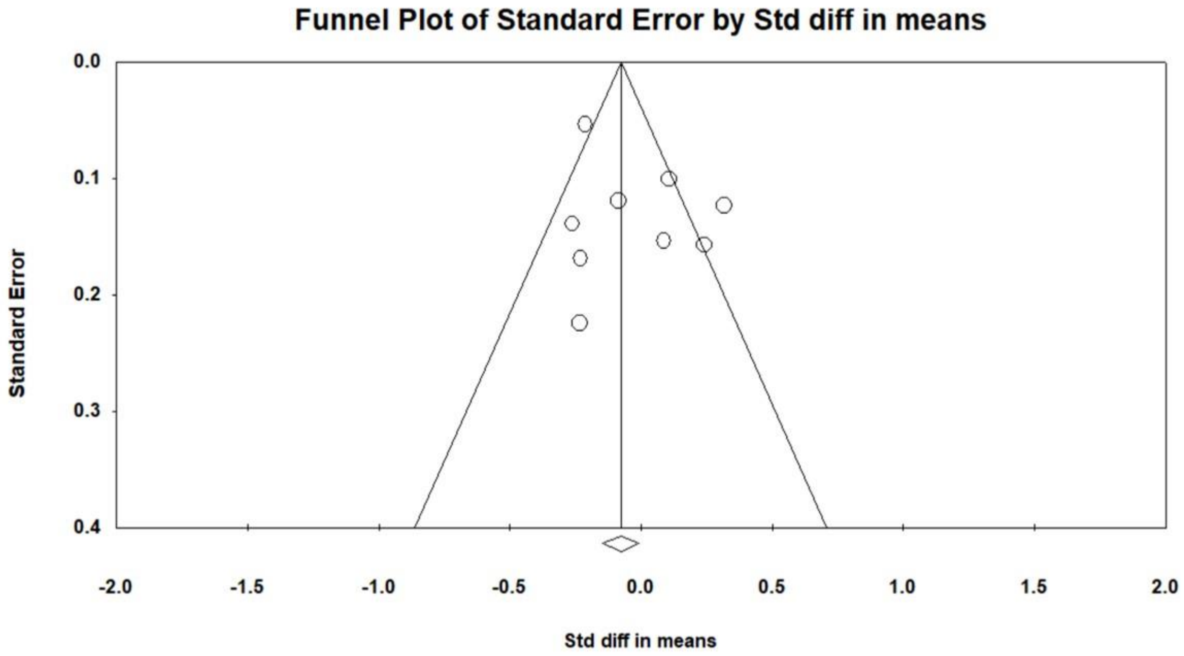

Figure 20.

The funnel plot showed non-significant publication bias according to Egger regression (*t*, 2.24; *df*, 2; *p* = 0.15).

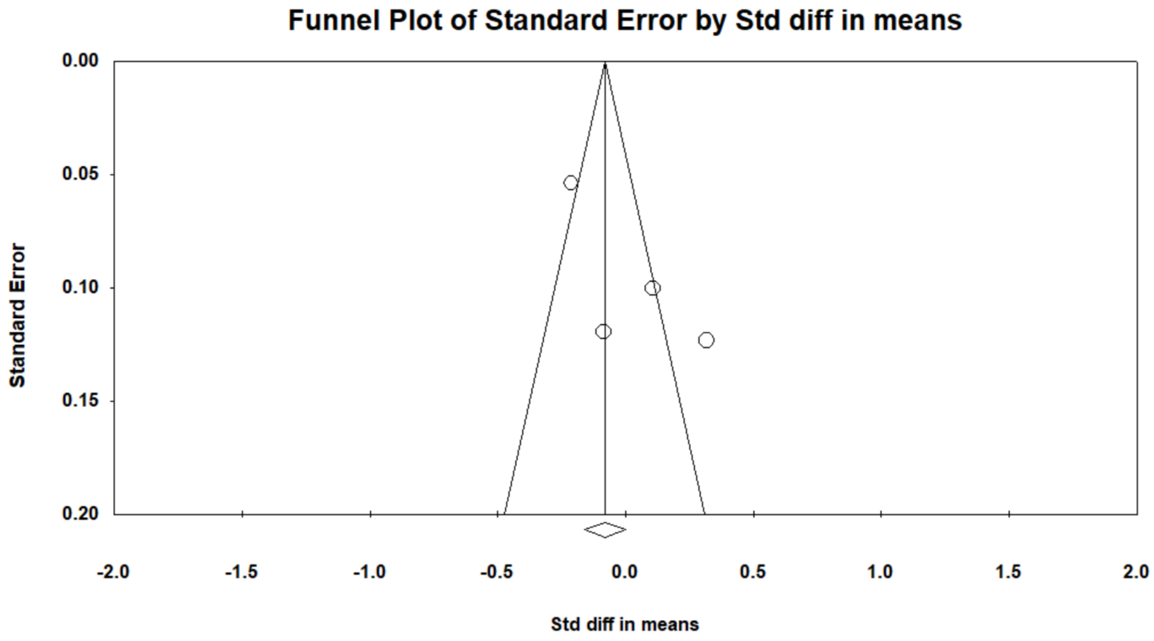

Figure 21.

The funnel plot showed non-significant publication bias according to Egger regression (*t*, 0.24; *df*, 3; *p* = 0.82).

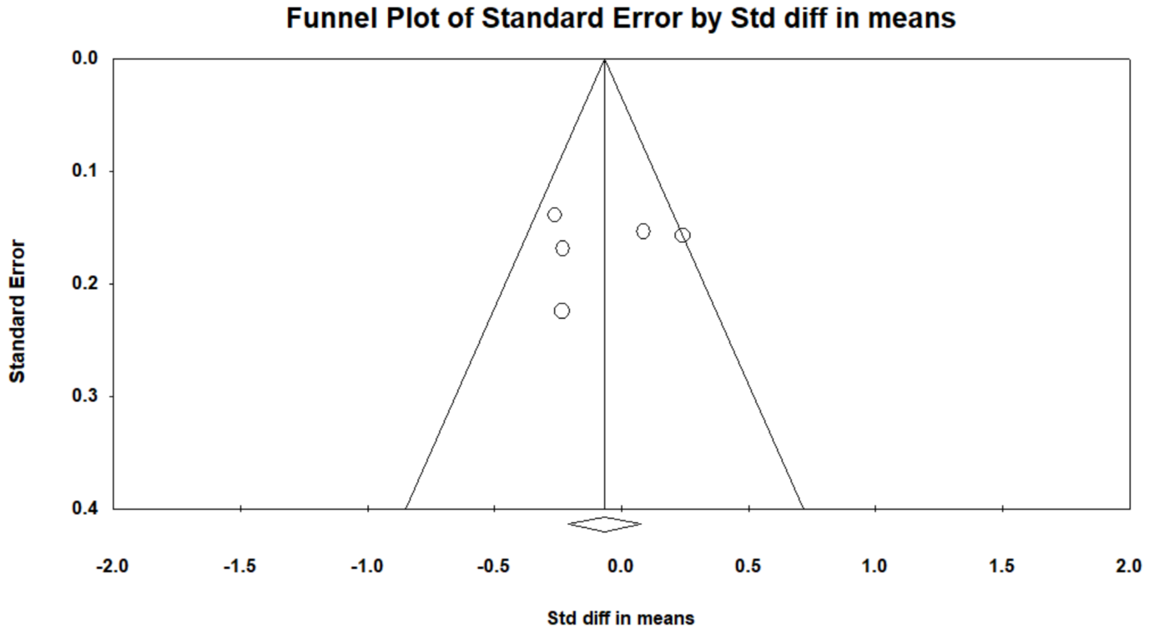

Funnel Plot of Standard Error by Std diff in means
Standard Error
0.0
0.1
0.2
0.3
0.4
-2.0
-1.5
-1.0
-0.5
0.0
0.5
1.0
1.5
2.0
Std diff in means
